# Host Defense and Recruitment of Foxp3^+^ T Regulatory Cells to the Lungs in Chronic *Mycobacterium tuberculosis* Infection Requires Toll-like Receptor 2

**DOI:** 10.1371/journal.ppat.1003397

**Published:** 2013-06-13

**Authors:** Amanda McBride, Jill Konowich, Padmini Salgame

**Affiliations:** UMDNJ-New Jersey Medical School, Department of Medicine, Centre for Emerging Pathogens Newark, Newark, New Jersey, United States of America; Weill Medical College of Cornell University, United States of America

## Abstract

Acute resistance to low dose *M. tuberculosis* (Mtb) infection is not dependent on Toll-like receptor (TLR) 2. However, whether TLR2 contributes to resistance in chronic Mtb infection has remained uncertain. Here we report that, following low dose aerosol infection with Mtb, mice lacking TLR2 (TLR2KO), in comparison with wild type (WT) mice, exhibit enhanced cellular infiltration and inflammation in the lungs, and fail to stably control bacterial burden during chronic infection. IFNγ and IL-17 was expressed at equivalent levels in the two groups; however, the characteristic accumulation of Foxp3^+^ T regulatory cells (Tregs) in pulmonary granulomas was significantly reduced in TLR2KO mice. Nonetheless, this reduction in Tregs was independent of whether Tregs expressed TLR2 or not. To directly link the reduced number of Tregs to the increased inflammation present in the TLR2KO mice, we used a macrophage adoptive transfer model. At seven weeks post-Mtb infection, TLR2KO mice, which were adoptively transferred with WT macrophages, displayed enhanced accumulation of Tregs in the lungs and a concomitant reduction in inflammation in contrast with control mice that received TLR2KO macrophages. However, the pulmonary bacterial burden between the two groups remained similar indicating that TLR2's role in modulating immunopathology is functionally distinct from its role in restricting Mtb growth in chronic infection. Together, these findings unequivocally demonstrate that TLR2 contributes to host resistance against chronic Mtb infection and reveal a novel role for TLR2 in mediating the recruitment of Foxp3^+^ Tregs to the lungs to control inflammation.

## Author Summary

Tuberculosis (TB) is an important cause of mortality in many parts of the world. Infection with *Mycobacterium tuberculosis* (Mtb), the causative agent of TB, is usually acquired via inhalation of airborne droplets containing the bacteria. Following inhalation, Mtb interacts with specialized receptors, called Toll-like receptors (TLRs), on phagocytic cells present in the lung. In this study, we examine the contribution of TLR2 in activating the body's natural defenses against Mtb. Wild type mice infected with Mtb by the aerosol route are able to control bacterial replication in the lung and maintain it at a steady level during chronic infection. However, in genetically modified mice that do not express TLR2 (TLR2KO), Mtb infection leads to increased inflammation in the lung and inability to control Mtb growth. Here, we identify that the increased inflammation present in the lungs of Mtb-infected TLR2KO mice is due to the diminished ability of a type of regulatory cell (Foxp3^+^ Tregs) to accumulate in the lungs. The ability to recruit Tregs to the lungs is restored in TLR2KO mice if they are adoptively transferred with macrophages from wild type mice. In summary, we demonstrate that TLR2 functions in protection against chronic Mtb infection by controlling Treg accumulation in the lung to limit inflammation and tissue damage.

## Introduction

Mtb expresses a large diversity of TLR2 ligands, including several types of lipoproteins and glycolipids, and also a trehalose dimycolate [1]–[3]. Interaction of these ligands with TLR2 expressed on macrophages and dendritic cells has multiple downstream effects. Several studies have reported that Mtb-derived TLR2 ligands produce a pro-inflammatory response [4]–[6], and consistent with these findings, mice deficient in TLR2 have diminished IL-17 response [7]. TLR2 signaling also induces direct antimicrobial activity in Mtb-infected human macrophages [8] by vitamin D3-dependent up-regulation of anti-microbial peptides [9]. Additionally, TLR2 signaling leads to suppressive effects on the functions of antigen-presenting cells (APCs). For example, TLR2 signaling in APCs induces IL-10 secretion [10] and prolonged signaling inhibits MHC class II expression [11], [12], antigen processing [3], [13], and IFNγ responsiveness [14].

Despite the extensive remodeling of macrophage functions following TLR2 signaling, TLR2-deficient mice are able to resist acute Mtb infection [15]–[17] and develop an appropriate secondary immune response [18] following a low dose aerosol infection. However, TLR2-deficient mice infected with a high dose of Mtb are more susceptible than WT to chronic infection and display an exaggerated immune inflammatory response, characterized by pneumonitis and enhanced cellular infiltration [15], [17]. These findings implicate a potential role for TLR2 in controlling inflammation during chronic infection.

Risk of developing tuberculosis has been shown to be associated with polymorphisms within the TLR2 gene, particularly within the TIR domain [19]–[21]. Analysis of a polymorphic guanine-thymine (GT) repeat located upstream of the TLR2 translational start site correlated shorter GT repeats with development of tuberculosis (TB) and lower TLR2 expression [22]. In addition, a TLR1 transmembrane domain polymorphism was shown to regulate the innate immune response to triacylated lipopeptides as well as extracts of mycobacteria [23]. Although the mechanism behind how these polymorphisms affect the immune response to Mtb is unclear, these correlations suggest an important role for TLR2 in host defense against Mtb. The aim of this study was to identify the mechanism by which TLR2 signals control inflammation and contribute to host resistance against Mtb. Here, we report that TLR2 functions in protection against chronic Mtb infection by keeping bacterial replication in check and limiting inflammation through recruitment of Foxp3^+^ Tregs to the lungs.

## Results

### Long term control of Mtb infection is compromised in the absence of TLR2

**Figure 1:**
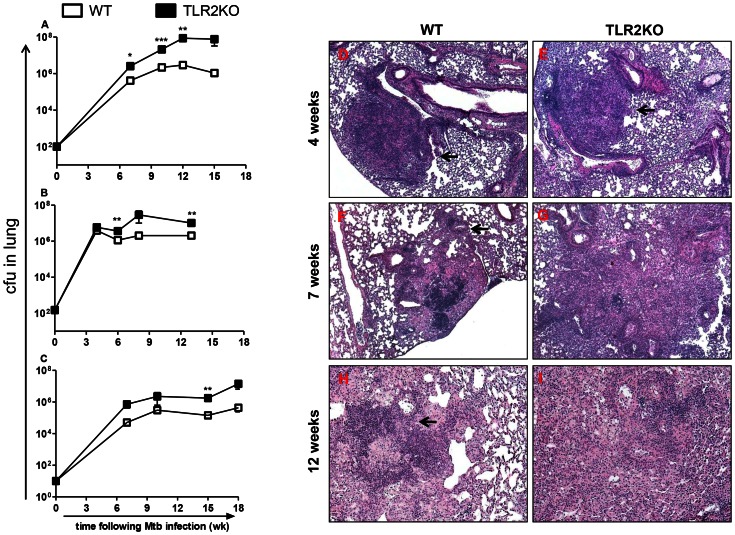
Loss of control of bacterial burden and disrupted granuloma morphology in the absence of TLR2 during chronic stages of Mtb infection. Bacterial burden in the lungs following aerosol infection with Mtb at doses of 100 CFU (A), 150 CFU (B), or 10 CFU (C) was compared between WT and TLR2KO mice. The numbers of viable bacilli were determined by plating serial dilutions of lung homogenates onto 7H11 agar plates. CFU were counted after 21 days of incubation at 37°C. Each time point includes 4–5 mice per group, except for the 15 week time point in (A), which includes 2 mice per group, and the 18 week time point in (C), which includes 4 WT and 3 KO mice. Panel (A) is representative for 1 of 3 independent experiments. Data are presented as mean CFU counts ± SEM. Asterisks represent statistically significant differences between WT and KO at a particular time point. * = p<0.05, ** = p<0.01, *** = p<0.001. Formalin-fixed, paraffin-embedded lung tissue was obtained at 4 weeks (D–E), 7 weeks (F–G), and 12 weeks (H–I) following infection. Sections were stained using a standard H&E protocol. Photomicrographs were taken at 5× (D–G) and 10× (H–I) magnification. Representatives of sections from 4–5 mice per group are shown.

We first evaluated the role of TLR2 in host resistance against chronic Mtb infection. WT and TLR2KO mice were aerosol-infected with approximately 100 CFU of Mtb and disease progression was monitored for 15 weeks. As shown in Figure 1A, TLR2KO mice exhibited a significantly increased bacterial burden at weeks 7 and 10, and, by week 12, there was more than a log increase in bacterial burden in the TLR2KO mice as compared with WT. Beginning at 10 weeks following infection, the TLR2KO mice also began to succumb to infection. The WT mice, as expected, were able to control infection and maintain a steady bacterial load. A repeat experiment using a similar infectious dose of Mtb (around 150 CFU) demonstrated consistent findings (Fig. 1B).

Previous studies have indicated that the magnitude of the immune response leading to control of Mtb infection can be dependent on infectious dose [24]. To ensure that the reduced resistance of TLR2KO mice was not dependent on Mtb inoculum size, disease progression in response to a very low inoculum was also followed. During aerosol infection with a low dose of Mtb (approximately 10 CFU), TLR2KO mice again demonstrated a log increase in pulmonary bacterial burden, although increased morbidity was not observed until around 18 weeks post-infection, at which point the difference in bacterial burden between WT and TLR2KO mice was around 2 logs (Fig. 1C). Consistent with the differences observed in bacterial loads, acid-fast staining of infected lung tissue demonstrated increased bacilli within lung macrophages in the TLR2KO mice (S1A). The absence of TLR2 did not affect Mtb dissemination and replication outside of the lung since differences were not observed in the spleen (S1B).

### Disrupted pulmonary granuloma architecture during chronic Mtb infection in the absence of TLR2

The development of pulmonary pathology in H&E stained sections of infected TLR2KO and WT mice was next characterized (Figure 1, D–I). The murine granulomatous lesion is a collection of peripheral lymphocytic aggregates with B cell follicles juxtaposing areas with macrophages and other inflammatory cells types. These lesions lack the heterogeneity exhibited by human granulomas; although the granulomatous reaction is progressive, necrosis is not exhibited until an exorbitant bacillary load is achieved, and caseous necrosis and cavitation do not occur in C57BL/6 mice [25]. At 4 weeks post-infection, tissue architecture was similar between both groups, with distinct areas of granulomatous cellular infiltration (arrows) surrounded by unaffected lung areas apparent in WT and TLR2KO (Figure 1, D and E). As infection progressed, the granuloma architecture in the TLR2KO began to deviate from what is normally observed in WT mice. At 7 weeks post-infection, WT mice developed typical compact granulomatous lesions (arrow) containing macrophages and lymphocytic infiltrates (Figure 1F). In comparison, lungs of the TLR2KO mice exhibited increased inflammation with disrupted granuloma architecture (Figure 1G). Similarly, during the chronic stage at 12 weeks post-infection, the characteristic granulomatous structure of macrophages and densely compact lymphocytes was apparent in the WT lungs (Figure 1H) while extensive cellular infiltration and markedly reduced alveolar spaces were observed in TLR2KO lungs (Figure 1 I). The TLR2KO lungs displayed poorly formed granulomas, with loosely aggregated lymphocytes dispersed amongst macrophages (Figure 1 I).

Overall, the TLR2KO mice exhibited an early inflammatory response to Mtb similar to WT. However, as the infection progressed towards the chronic stage, the granuloma architecture in the WT lungs stabilized, while the TLR2KO lungs became consolidated with infiltrates which disrupted the granuloma morphology and progressively spread to comprise a majority of the lungs. These data confirm and extend the findings reported by Drennan and colleagues [17] that Mtb infection of TLR2KO mice leads to an exaggerated inflammatory response in the lungs.

Flow cytometric analysis of the cell surface markers CD11c, CD11b, and Gr-1 demonstrated significant increases in the numbers of CD11c^−^CD11b^+^ recruited macrophages, CD11c^+^CD11b^+^ myeloid DCs, CD11c^+^CD11b^−^ alveolar macrophages, and Gr-1^hi^CD11b^+^ neutrophils infiltrating the lungs at a late stage of infection (10 and 13 weeks) (Figure S2B). Differences in NK1.1^+^ NK cells and CD19^+^ B cells were not observed (data not shown). Overall, these observations of increased recruitment of inflammatory cells to the lungs in the absence of TLR2 are consistent with the severe inflammatory lung pathology in TLR2KO mice during late stages of infection.

### Decreased CD4^+^Foxp3^+^ regulatory T cells in the lungs during Mtb infection in the absence of TLR2

**Figure 2:**
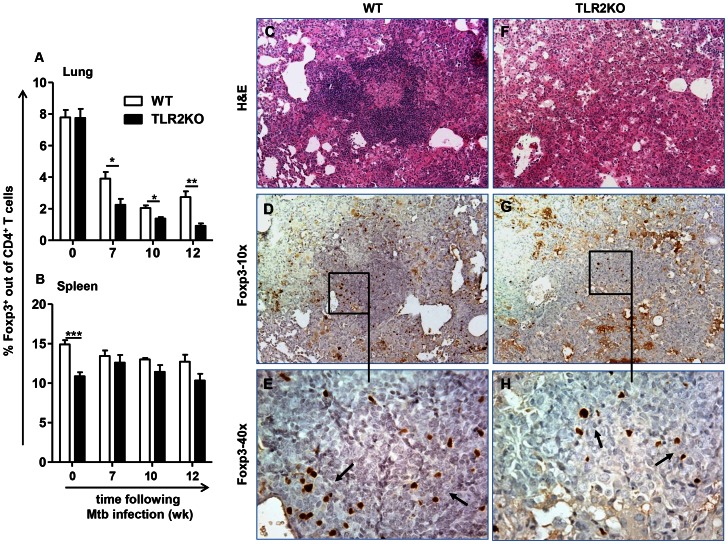
Decreased natTregs observed in the lungs in the absence of TLR2. Lungs and spleens were harvested from WT and TLR2KO mice, and single cell suspensions were prepared at the indicated time points after Mtb challenge. Cells were stained with antibodies against CD4, followed by intracellular staining for Foxp3. Expression of Foxp3 is presented as percentage of the CD4^+^ cell population in lung (A) and spleen (B). 11 mice per group were included for uninfected (week 0 = uninfected). Each time point post-infection (weeks 7, 10, and 12) represents 5 mice per group. Post-infection Treg analysis shown is representative of 5 independent experiments. Data are presented as mean ± SEM. * = p<0.05, ** = p<0.01, *** = p<0.001. Formalin-fixed, paraffin-embedded lung tissue was obtained at 12 weeks following infection. Serial sections showing areas of granulomatous inflammation in WT (C–E) and TLR2KO (F–H) mice are shown. Sections were stained with H&E (C and F) or with anti-Foxp3 (D, E, G and H). In sections stained with anti-Foxp3, areas within inflammatory lesions (D and G) are shown at higher magnification (E and H). Brown pinpoint staining indicative of Foxp3^+^ staining was not observed in serial sections stained with isotype control (not shown). Photomicrographs were taken at 10× (C, D, F, and G) and at 40× (E and H) original magnification. Sections are representative of 5 mice per group.

The enhanced inflammatory response in the lungs of Mtb infected TLR2KO mice led to the hypothesis that a regulatory T cell population may be lacking in the absence of TLR2. Natural Foxp3-expressing regulatory T cells (natTregs) are present in Mtb granulomas [26]. Therefore, the presence of natTregs in the lungs based on expression of Foxp3 was investigated. Lungs were harvested at the indicated time points post-infection, and flow cytometric analysis was performed to determine the percentage of Foxp3^+^ cells out of the CD4^+^ T cell population (Figure S3). The percentage of CD4^+^ cells expressing Foxp3 in the lungs was similar between WT and TLR2KO mice prior to Mtb infection. However, following infection, the TLR2KO mice displayed decreased frequencies of Foxp3^+^ Tregs in the lungs compared to WT (Figure 2A). While the percentage of Foxp3^+^ cells was lower in TLR2KO lungs than WT lungs, Tregs in both groups had equivalent expression of glucocorticoid-induced TNF receptor-related protein (GITR) and cytotoxic T-lymphocyte antigen-4 (CTLA-4), indicating a true natTreg phenotype of the Foxp3^+^ cells present in TLR2KO lungs (data not shown). Of note, it was also observed that both groups displayed decreased percentages of Foxp3^+^ cells out of the CD4^+^ population in the lungs compared to their naïve counterparts, although to a greater extent in the TLR2KO. This decrease is probably reflective of a greater expansion of CD4^+^ effector T cells compared to CD4^+^ Tregs following Mtb infection. Enumeration of cell infiltrates showed that total cell numbers, and CD4^+^ and CD8^+^ T cell numbers in TLR2KO mice were similar to WT at 7 weeks following infection (Figure S2A), a time when the percentage of Treg cells was lower in TLR2KO. This supports that the decreased percentage of Tregs in TLR2KO mice is not merely due to the greater expansion of T effector cells.

However, total cell numbers and T cell numbers continually increased over time in TLR2KO mice, while they stabilized in WT mice (Figure S2A). Therefore, we further investigated differences in Foxp3^+^ cells in the WT and TLR2KO by immunohistochemical staining of lung sections for the presence and localization of Foxp3-expressing cells. At 4 weeks post-infection, there were few Foxp3-expressing cells present in the granulomatous lesions of either WT or TLR2KO mice, although more were apparent in WT. At this time point, Foxp3^+^ cells were present in perivascular and peribronchiolar regions in both groups (Figure S4, Panels G and H). By 12 weeks post-infection, examination of WT (Figure 2C) and TLR2KO lung lesions (Figure 2F) by immunochemistry showed accumulation of high numbers of Foxp3^+^ cells in lesions of WT mice (Figure 2D). In contrast, very few Foxp3^+^ cells were observed in the affected areas of the lungs of TLR2KO mice at this time point (Figure 2G). Individual Foxp3^+^ cells or small clusters of Foxp3^+^ cells were dispersed randomly in TLR2KO lungs (Figure 2H), although they were not aggregated to the same extent as in WT (Figure 2E). Together, these findings show that TLR2 signals are necessary for the accumulation of regulatory T cells in the lungs during Mtb infection.

**Figure 3:**
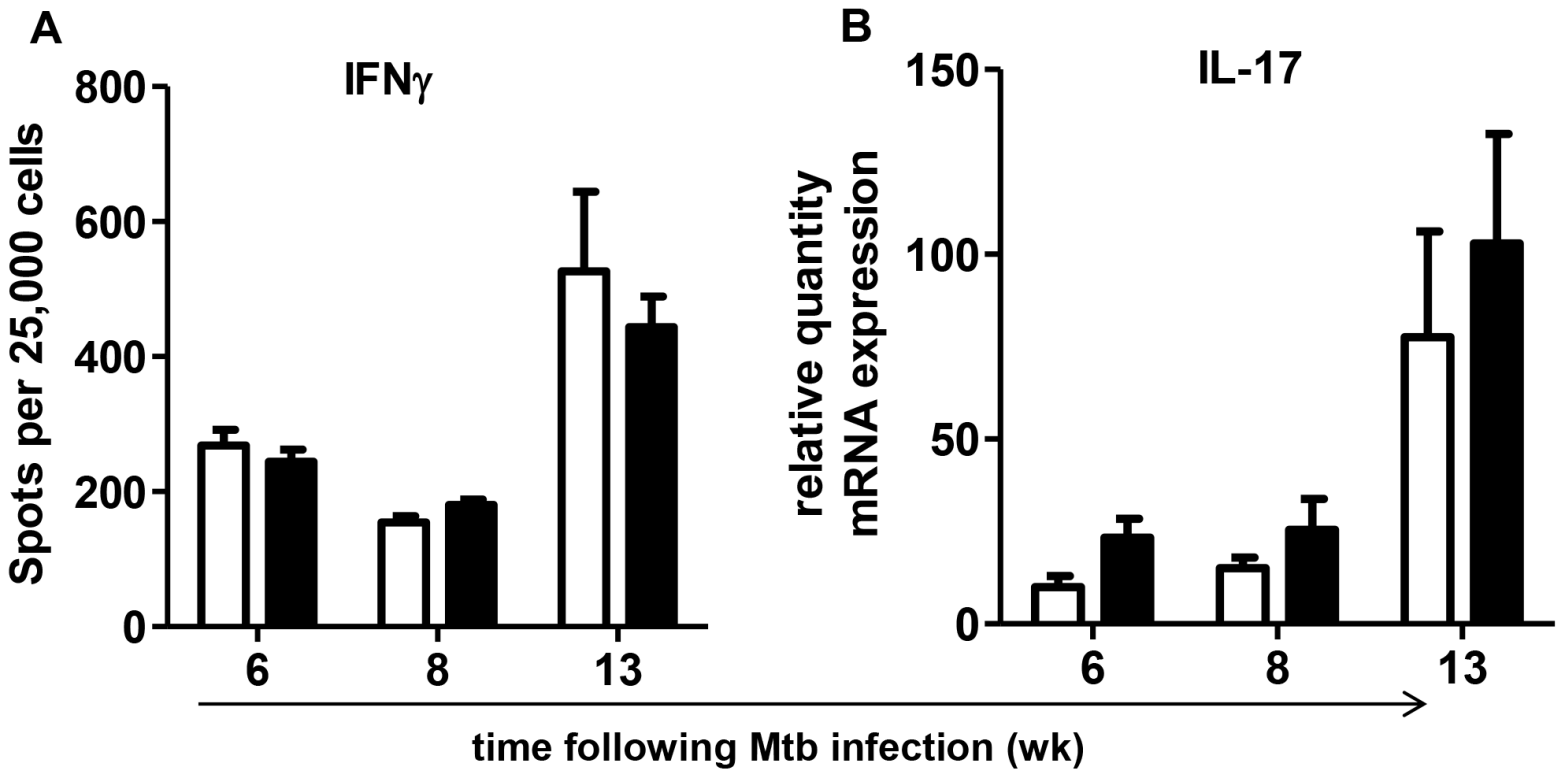
Similar IFNγ and IL-17 levels in WT and TLR2KO Mtb-infected mice. Single cell suspensions from lungs of WT and TLR2KO mice were re-stimulated overnight with Mtb-pulsed WT bone marrow-derived dendritic cells and the frequencies of Mtb-specific IFNγ-producing cells were determined by ELISPOT (A). Expression levels of IL-17 (B) in the lungs of WT and TLR2KO mice during Mtb infection were determined by isolation of total lung RNA followed by real time RT-PCR analysis. Each time point in A and B includes 4–5 mice per group, and data are representative of two independent experiments. All data are presented as mean ± SEM. No significant differences were observed between the WT and TLR2KO mice.

Other studies using murine models of Mtb infection have demonstrated that depletion of CD4^+^CD25^+^Foxp3^+^ natTregs or the complete absence of this population results in increased frequencies of IFNγ-producing CD4^+^ effector T cells [27], [28]. Given the importance of IFNγ in the protective immune response against Mtb, the possibility that decreased frequencies of Foxp3^+^ Tregs in the lungs of TLR2KO mice may correlate to enhanced Th1 responses was investigated. ELISPOT assay was performed with cells isolated from the lungs during chronic stages of infection and re-stimulated with Mtb-pulsed bone marrow-derived DCs serving as APCs. As shown in Figure 3A, there were no differences in the numbers of Mtb-specific IFNγ secreting cells in the lungs of WT and TLR2KO mice. Further, the decreased accumulation of Foxp3^+^ Tregs did not correlate to enhanced IL-17 gene expression, as there were no significant differences in IL-17 gene expression between WT and TLR2KO lungs (Figure 3B). Overall, these results indicate that the decreased accumulation of Foxp3^+^ Tregs in the lungs of TLR2KO mice is not associated with enhanced Mtb-induced Type I T cell responses.

### TLR2 signaling on CD4^+^Foxp3^+^ T cells is not required for their recruitment to the lungs during Mtb infection

It has been reported that TLR2 signaling on natTregs promotes their expansion and survival [29]–[31]. Therefore, the decreased number of Foxp3^+^CD4^+^ cells observed in the lungs of TLR2KO mice could result from decreased expansion of this population in peripheral lymphoid organs. To address this, the levels of Foxp3^+^ Tregs in the spleens were monitored following Mtb infection. Consistent with a previous report [32], decreased Foxp3^+^CD4^+^ cells were observed in the spleens of naïve TLR2KO mice as compared to WT levels. However, during Mtb infection this difference was no longer observed (Figure 2B), suggesting that differences in peripheral expansion of Foxp3^+^ Tregs between WT and TLR2KO mice do not account for the decreased frequencies of these cells in the lungs in the absence of TLR2.

**Figure 4:**
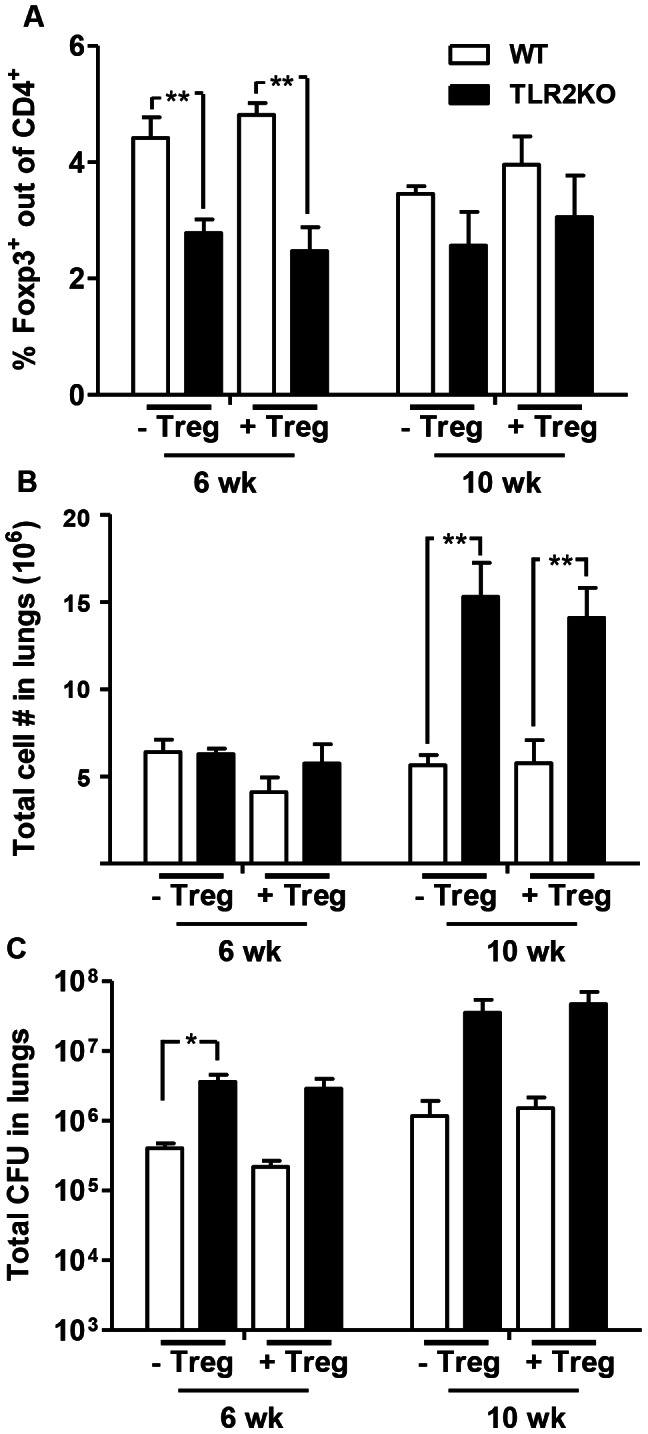
Treg accumulation and disease progression in TLR2KO mice is not altered by the transfer of WT Tregs. CD4^+^CD25^+^ Tregs purified from naïve WT mice were transferred to TLR2KO and WT control mice one day prior to and 4 weeks after Mtb infection (0.5×10^6^ cells per mouse for each transfer). Non-recipient WT and TLR2KO control mice were infected at the same time. Single cell suspensions were prepared at the indicated time points after Mtb challenge. Lung cells were stained with antibodies against CD4 and Foxp3, followed by flow cytometric analysis. Expression of Foxp3 is presented as a percentage of the CD4^+^ cell population (A). Total number of viable cells in the lungs was determined by trypan blue exclusion method (B). Bacterial burden in the lungs was determined by plating serial dilutions of lung homogenates onto 7H11 agar plates (C). Data include 4–5 mice per group and are presented as mean ± SEM. _*,_ p<0.05; _**_, p<0.01.

Given the possibility that TLR2 signals are important for natTreg survival, we determined if reconstitution of TLR2KO mice with WT Tregs would allow for Treg accumulation in TLR2KO lungs. CD4^+^CD25^+^ Tregs from naïve WT mice were transferred to both TLR2KO and WT mice one day prior to and 4 weeks post-Mtb infection. TLR2KO and WT mice that did not receive Tregs were infected at the same time as controls. Flow cytometric analysis of Foxp3^+^CD4^+^ cells at 6 and 10 weeks post-infection indicated that the transfer of WT Tregs did not increase the frequency of this population in the lungs of TLR2KO mice, which were significantly lower than WT controls at 6 weeks (Figure 4A). Consistent with this, total pulmonary cell numbers at 10 weeks were significantly increased in both recipient and non-recipient TLR2KO as opposed to WT controls (Figure 4B). Also, the transfer of Foxp3^+^Tregs did not affect bacterial burden in the lungs. Total CFU in the lungs was increased by 1 log in the TLR2KO groups at both 6 and 10 weeks, although this increase was only significant at 6 weeks within the non-recipient group (Figure 4C). Together, these data demonstrate that the transfer of WT Foxp3^+^ Tregs failed to restore Treg numbers to WT levels in the lungs of TLR2KO hosts.

To definitively address whether the absence of TLR2 on natTregs is responsible for their decreased accumulation in the lungs of Mtb-infected TLR2KO mice, an adoptive transfer model using T cell-deficient Rag2^−/−^ mice was used (described in Figure S5A). Conventional naïve CD4^+^CD25^−^ T cells (which will be referred to as Tconv) and CD4^+^CD25^+^ T cells (Treg) were purified from WT (CD45.1^+^) and TLR2KO (CD45.2^+^) mice. By flow cytometry, greater than 99% of sorted CD25^+^ cells reacted with anti-Foxp3 antibody (data not shown). Rag2^−/−^ mice were reconstituted one day prior to Mtb infection with combinations of 2×10^6^ Tconv cells and 2×10^5^ Treg cells. Group I received WT Tconv (CD45.1) and TLR2KO Treg (CD45.2), and Group II received TLR2KO Tconv (CD45.2) and WT Treg (CD45.1). These combinations allowed for investigation of the effects of TLR2 signaling on Tconv and Treg populations separately, on an otherwise WT background for myeloid and stromal cells. At 4 and 9 weeks post-infection, Treg accumulation and bacterial burden was analyzed.

**Figure 5:**
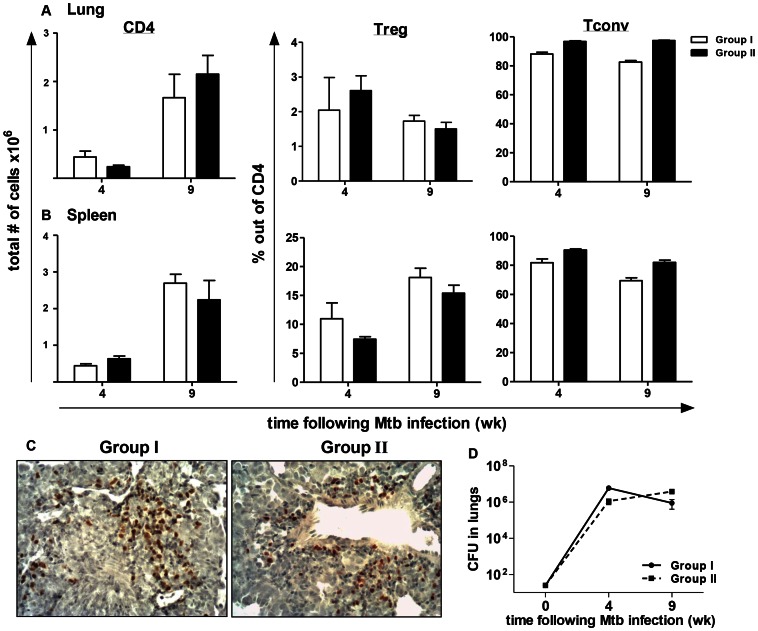
TLR2 expression on regulatory T cell populations does not affect their recruitment to the lungs. Single cell suspensions from lungs and spleens of mice from Groups I and II were prepared at the indicated time points after Mtb challenge. Lung and spleen cells were stained with antibodies against CD4, CD45.1, and CD45.2 and then analyzed by flow cytometry. For both lung (A) and spleen (B) samples, lymphocytes were gated on, and then CD4^+^ cells were gated from this population. The number of CD4^+^ cells was determined by multiplying the percentage of gated cells by total lung cell number (left panel). Frequencies of Treg (middle panel) and Tconv (right panel) were determined by gating on CD45.1 and CD45.2 populations within the CD4^+^ gate and are presented as a percentage out of CD4^+^. Formalin-fixed, paraffin-embedded lung tissue was obtained at 9 weeks following infection. Sections from Groups I and II were stained with anti-Foxp3 and photomicrographs were taken at 20× original magnification. Sections are representative of 6 mice per group (C). Bacterial burden in the lungs was determined by plating serial dilutions of lung homogenates onto 7H11 agar plates (D). Data include 6 mice per group and are presented as mean ± SEM. No significant differences were observed between the two groups for all the tested parameters.

Single cell suspensions were prepared from the lungs and spleens derived from the two groups of mice at the indicated time points after Mtb challenge and stained with antibodies against CD4, CD45.1, CD45.2 and Foxp3 for flow cytometric analysis. Lymphocytes were gated on, followed by gating on CD4^+^ cells. For Group 1, the frequencies of Treg and Tconv were determined by gating on CD45.2 and CD45.1 populations, respectively, within the CD4^+^ gate, and are presented as percentage out of CD4^+^ T cells. Similarly, for Group II, the frequencies of Treg and Tconv were determined by gating on CD45.1 and CD45.2 populations, respectively, within the CD4^+^ gate. The dot plot analysis for lung and spleen is presented in Supplementary Figure 5B and 5C, respectively. Equivalent numbers of CD4^+^ cells were recruited to the lungs in the two groups of mice at both 4 and 9 weeks post-infection (Figure 5A). Similarly, analysis of the Treg and Tconv populations out of the CD4^+^ cells showed that both groups had similar frequencies of these populations in the lungs (Figure 5A). Therefore, accumulation of Tregs in Group I mice that received TLR2KO CD4^+^CD25^+^ T cells was similar to that of Group II mice that had received WT CD4^+^CD25^+^ T cells. Analysis in the spleens showed a similar result as in the lungs, with no differences observed in CD4^+^ cell numbers or in the frequencies of Tregs between the two groups (Figure 5B). Immunohistochemistry for Foxp3^+^ cells also demonstrated that Foxp3^+^ Tregs could be detected in the lungs in both groups (Figure 5C). Consistent with the equivalent Treg/Tconv frequencies observed, Mtb growth kinetics in the lungs were comparable in both groups of mice (Figure 5D). Further flow cytometric analysis of the CD45.1 and CD45.2 populations in the lungs and spleen (Figure S5D–G) of the two groups of mice demonstrated that, in both groups, Foxp3 expression was retained at a similar level and was limited to the congenic marker of the original CD4^+^CD25^+^ injected population. These findings confirm that the Tregs which accumulated in the lungs of Group I originated from the TLR2KO CD4^+^CD25^+^ T cells injected and were not due to conversion of the injected WT CD4^+^CD25^−^ population. It is important to note that the immunoregulatory functions of B cells [33] are lacking in the reconstituted Rag2^−/−^ mice. Nonetheless, the findings from this experiment demonstrate that TLR2 signaling on CD4^+^Foxp3^+^ Tregs is not necessary for their expansion and subsequent recruitment into Mtb-infected lungs.

### Restoration of Foxp3^+^ Treg accumulation and the granulomatous response in the lungs of TLR2KO mice by WT macrophages

**Figure 6:**
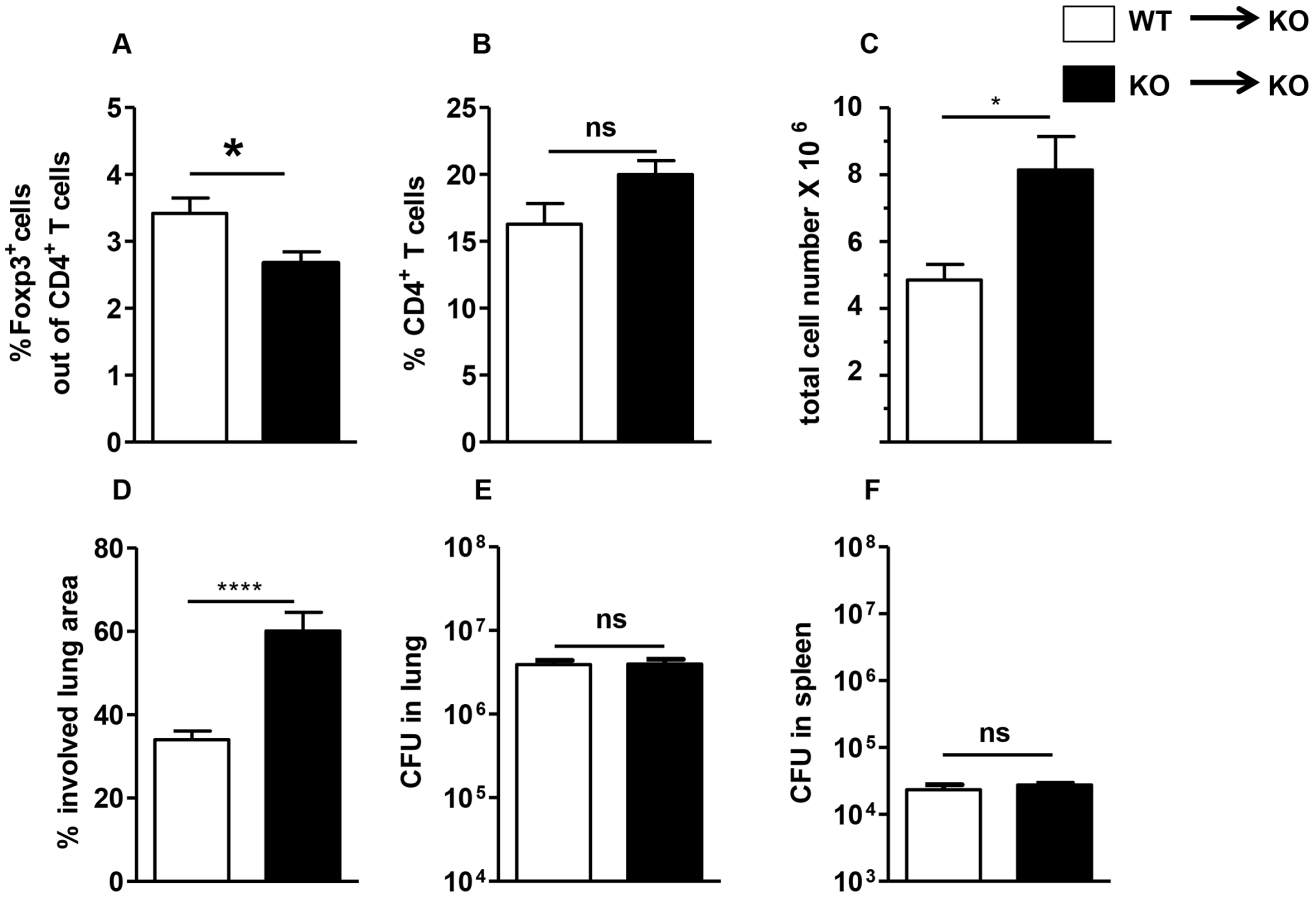
Rescue of WT phenotype in TLR2KO mice following intra-tracheal instillation of WT macrophages. TLR2KO mice were intra-tracheally instilled with 2.5×10^6^ WT peritoneal macrophages (WT→KO) or TLR2KO peritoneal macrophages (KO→KO) one day prior to infection with a low-dose of Mtb. Single cell suspensions were prepared at week 7 post-Mtb infection. Lung cells were stained with antibodies against CD4 and Foxp3, followed by flow cytometric analysis. Expression of Foxp3 is presented as a percentage of the gated CD4^+^ cell population (A). Percentage of CD4^+^ cells was determined by gating on CD4^+^ cells from total lymphocytes gated (B). Total number of viable cells in the lungs was determined by trypan blue exclusion method (C). Percentage of involved lung area was quantitated by superimposing a grid overlay onto photomicrographs of H&E stained lung sections taken with a 5× objective lens. One representative section per mouse was used for quantitation (D). Bacterial burden in the lungs (E) and spleen (F) was determined by plating serial dilutions of tissue homogenates onto 7H11 agar plates. Data include 5–6 mice per group and are presented as mean ± SEM. _*,_ p<0.05; _****_, p<0.0001.

**Figure 7:**
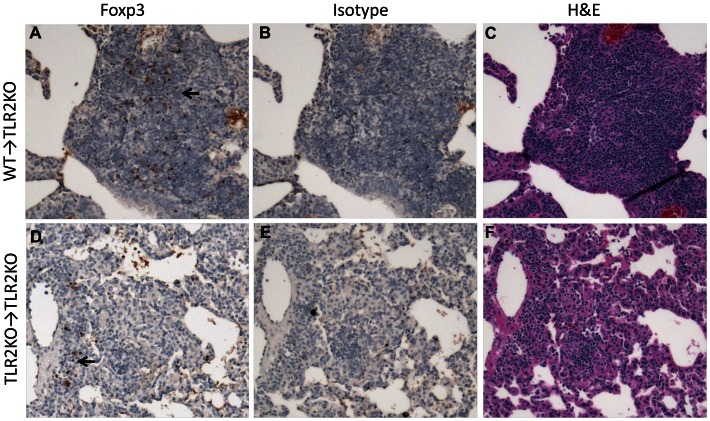
Histological evaluation displays increased accumulation of Tregs in WT → TLR2KO hosts is associated with reduced inflammation. Formalin-fixed, paraffin-embedded lung tissue was obtained at 7 weeks post-Mtb infection. Serial sections showing areas of granulomatous inflammation in WT→TLR2KO (A–C) and TLR2KO→TLR2KO (D–F) mice are shown. Sections were stained with anti-Foxp3 (A and D), isotype control (B and E), or H&E (C and F). Brown pinpoint staining indicative of Foxp3^+^ staining was not observed in serial sections stained with isotype control. Photomicrographs were taken at a 10× magnification (A–F). Representative sections are shown.

Since TLR2 signaling on Tregs themselves does not affect their accumulation into Mtb-infected lungs, we considered that TLR2 signals on pulmonary myeloid cells may play a role in Treg recruitment to granulomatous areas. To directly address this potential role of TLR2 on myeloid cells, we investigated whether providing TLR2-expressing WT myeloid cells to a TLR2KO host would restore normal accumulation of Foxp3^+^ Tregs in the lung and protect from inflammatory pathology. Macrophages purified from WT or TLR2KO mice were adoptively transferred directly into the lungs by intra-tracheal instillation one day prior to Mtb infection. At seven weeks post-infection, lungs from the TLR2KO mice adoptively transferred with WT macrophages (WT→TLR2KO mice) or TLR2KO macrophages (TLR2KO→TLR2KO mice) were harvested and evaluated for Treg accumulation, cellular infiltration, granulomatous inflammation, and bacterial burden. Flow cytometric analysis of single cell suspensions of lungs showed a significantly higher percentage of Foxp3^+^ Tregs in the WT→TLR2KO mice than the TLR2KO→TLR2KO mice (Figure 6A) while the percentage of CD4+ T cells was similar in the two groups (Figure 6B). Immunohistochemical staining of lung sections demonstrated high levels of Foxp3^+^ cell accumulation in the granulomatous lesions of WT→TLR2KO mice (Figure 7A), while very few Foxp3^+^ cells were observed in the affected areas of the lungs of TLR2KO→TLR2KO mice (Figure 7D). In the latter group, the sparsely recruited Foxp3^+^ cells were mainly observed in perivascular and peribronchiolar regions (Figure 7D). No background was observed in serial sections stained with isotype control (Figure 7B and E). Consistent with higher Foxp3^+^ Treg cell accumulation, WT→TLR2KO mice exhibited significantly less cellular infiltration than the TLR2KO→TLR2KO mice (Figure 6C). Histopathological evaluation of lung tissue revealed that the characteristic granulomatous structure with compact aggregation of cells observed in WT mice was restored in the WT →TLR2KO mice (Figure 7C), while lung tissue from the TLR2KO→TLR2KO mice exhibited loosely aggregated lymphocytes and increased inflammation (Figure 7F) with a significantly greater area of lung involvement as expected in a TLR2KO host (Figure 6D). While WT→TLR2KO mice exhibited improved Treg accumulation and decreased inflammatory pathology, the bacterial burden in the lungs (Figure 6E) and spleen (Figure 6F) was similar in both groups, indicating that the role of TLR2 in controlling bacterial burden may be distinct from its role in controlling inflammation. Overall, these findings indicate that TLR2 signaling from macrophages promotes Treg recruitment to the lungs and decreases inflammatory pathology during Mtb infection.

## Discussion

In this study, we conclusively demonstrate that Mtb infection in the absence of TLR2 results in poorly formed granulomas, progressive pulmonary pathology, and increased lung bacterial burden during chronic infection. Our data also indicate that Treg dysfunction may, in part, underlie the immune pathogenesis observed in the lungs of TLR2KO mice. Moreover, we have demonstrated that transfer of WT macrophages significantly enhanced the accumulation of Foxp3^+^ Tregs within pulmonary granulomatous lesions in TLR2KO mice and concomitantly alleviated pulmonary inflammation. This clearly establishes a causal role for Tregs in controlling the immunopathology of TB. Finally, our data that bacterial burden in the lungs of TLR2KO mice was not affected by the transfer of WT macrophages suggests that the immunoregulatory function of TLR2 can be uncoupled from its antibacterial function. TLR2 ligation activates a multitude of MAPK signaling pathways [34]; it is likely that these distinct pathways regulate the dual functions of TLR2 in chronic infection.

The finding that Tregs expand independently of direct TLR2 signals was surprising given the evidence that TLR2 ligation on Foxp3^+^ Tregs enhances their proliferation and survival [29]–[31]. These prior reports, however, investigated the effects of TLR2 signaling on Tregs through direct ligation by synthetic TLR2 ligands, not in the context of TLR2 signaling that may occur during an infection. Our study demonstrates that, during Mtb infection, TLR2 signaling on Tregs does not significantly contribute to the expansion and recruitment of Tregs to sites of infection. Instead, TLR2 activation on myeloid cells is necessary to induce the accumulation of Tregs in the lungs. The precise mechanism by which myeloid cells contribute to Treg accumulation remains to be determined, but it is likely dependent on the initiation of an appropriate chemokine axis in the microenvironment of the granuloma. For example, Tregs express the chemokine receptor CCR4 and are highly chemotactic towards its ligands, macrophage-derived chemokine (MDC/CCL22) and thymus and activation regulated chemokine (TARC/CCL17) [35], [36]. While the secretion of CCL22 and CCL17 has been implicated in Treg recruitment to the tumor microenvironment in several studies [37]–[41], the role of these chemokines in the recruitment of Tregs to inflammatory sites during Mtb infection has yet to be addressed. It has also been shown that a subset of Foxp3^+^ Tregs expressing the Th1 transcription factor T-bet as well as the chemokine receptor driven by T-bet, CXCR3, accumulate at sites of Th1-mediated inflammation [42]. Therefore, a deficiency in any of the IFNγ-inducible CXCR3 ligands may affect Treg recruitment to Mtb granulomas. It is also possible that activation of TLR2 on macrophages may be important for supporting Treg proliferation and maintenance in the lung following recruitment. Future experiments using specific antibodies and gene-deficient mice are necessary to analyze these various possibilities.

Mycobacterial lipids, including TLR2 ligands such as LpqH, are released from Mtb-infected macrophages via exocytosis [43], [44]. Also, Mtb releases membrane vesicles within macrophages that stimulate cytokine and chemokine release in a TLR2-dependent fashion [45]. Therefore, it is conceivable that during chronic infection, Mtb-derived TLR2 ligands are released into the granuloma microenvironment where they can interact with macrophages and perhaps dendritic cells to initiate the chemokine axis required to direct Tregs towards areas of granulomatous inflammation in the lungs and, subsequently, into the granuloma. Our findings indicate that Tregs are necessary to control granulomatous inflammation and maintain a stable granuloma. However, other studies found that natTregs undergo expansion in the blood and at disease sites, and their removal from circulation improved cytokine production from T cells [46]–[48]. Also, Tregs were shown to delay Th1 cell activation in the murine model [49]. Together, these data suggest that the temporal removal of Tregs may be beneficial to the host in enhancing a protective immune response, but, because of the persistent nature of Mtb, it is critical that sufficient numbers of Tregs are recruited to the lungs to mitigate immunopathology. This idea is supported by studies in non-human primates which found that higher frequencies of Tregs correlated to the development of latent TB over active TB disease [50] and that Tregs and Teffector cells acted together to control inflammation without enhancing Mtb replication [51].

The current findings that TLR2 is required for controlling chronic Mtb infection support and extend a previous study [17]. However, these results contradict studies by Holscher and colleagues who reported that TLR2/4 double- and TLR2/4/9 triple-deficient mice are able to efficiently control low dose aerosol infection with Mtb [52], [53]. Reasons for these variable outcomes to Mtb infection are not clear, but they could be related to different experimental conditions, dose of infection, Mtb strain, or perhaps to differences in commensal microbiota. A recent study by Iwasaki and colleagues [54] showed that gut microbiota, critical for maintaining immune homeostasis in the gut mucosa [55], [56], can also influence immunity to infection at a distant site, such as the respiratory mucosa. The authors demonstrated that the commensal microbiota were required for optimal activation of the adaptive immune response against influenza virus infection by providing signal 1 for the expression of mRNA for pro–IL-1β and pro–IL-18 at steady state. This requirement for intact commensal bacteria to generate an appropriate adaptive immune response was found to be restricted to pathogens that are dependent on inflammasomes for immune cell priming, and not to all respiratory pathogens. For example, T cell and B cell responses to *Legionella pneumophila* were not affected in antibiotic-treated animals. Given that NLP3 inflammasome activation is linked to exacerbated pathology in the lungs of Mtb-infected mice [57], it is possible that microbiota may differentially influence the outcome of Mtb infection in mice bred at different facilities. Our finding that the transfer of WT macrophages restores Treg accumulation and results in improved control of inflammation in TLR2KO mice reduces the likelihood that our observations were influenced by differences in the microbiota of WT and TLR2KO mice that were bred at different sites. Nevertheless, it does not rule out the possibility that differences in microbiota could be a contributing factor to the discrepant results seen between our studies and those of Holcher and colleagues [52]. Depending on the composition of the microbiota, the steady state expression of pro-IL-1β and IL-18 may vary, thus necessitating differences in the requirement of regulatory components to control pulmonary inflammation during Mtb infection. This is an area rich for future investigations.

There are several possible pathways that may be activated by Tregs to prevent immunopathology and associated tissue damage. There is now accumulating evidence to indicate that beyond its anti-microbial function, IFNγ also limits immunopathology in Mtb-infected hosts. IFNγ down-modulates IL-17 production and the subsequent accumulation of pathogenic neutrophils [57]. IFNγ signalingalso dampens the production of the pro-inflammatory cytokines, IL-1α and IL-1β, from myeloid cells [58], via NOS2-mediated inhibition of assembly and activation of the NLRP3 inflammasome [59]. Conspicuous B cell aggregates with characteristic germinal center features are present in the lungs of Mtb-infected mice [60] and emerging evidence indicates a regulatory role for these cells during Mtb infection. Mtb-infected mice deficient in B cells display an exaggerated immunopathology associated with enhanced neutrophil recruitment to the lungs [33]. The enhanced inflammation observed in the TLR2KO mice in our study, however, was not associated with alterations in IFNγ-secreting cells, IL-17 gene expression, or B cell numbers during chronic infection. Despite this, the increased mononuclear cell infiltration suggests that Tregs may function to restrain the influx of monocytes and neutrophils to the lung. Although neutrophils provide protection during acute infection [61], their presence in the lung in chronic infection is associated with pathology ([62] and reviewed in [63]). Thus, by controlling neutrophil recruitment, Tregs may limit inflammation and pulmonary pathology. Also, it is possible that by regulating cellular recruitment, Tregs serve to limit the availability of intracellular niches for Mtb to replicate. Indeed, inhibition of recruitment of new macrophages to the granuloma reduces Mtb numbers in the lung [64], [65]. Although we have delineated that TLR2 controls Mtb growth and inflammation via distinct mechanisms, it is possible that an interplay could exist between the two mechanisms. Future studies will address whether the increased bacterial burden observed in TLR2KO hosts is due to the absence of TLR2-mediated antimicrobial activity within the granuloma or as a consequence of the enhanced inflammation. In sum, our study supports that the TLR2/Treg axis is one of several regulatory circuits that are activated during chronic Mtb infection to safeguard the host against exaggerated inflammation and damage induced by the persistence of Mtb in the lungs.

## Materials and Methods

### Ethics statement

All animal experiments described in this study conform with the UMDNJ Newark IACUC Guidelines, NIH and USDA policies on the care and use of animals in research and teaching, and the policies of the Guide for the Care and the Use of Laboratory Animals. Animal protocols used in this study were approved by the UMDNJ Institutional Animal Care and Use Committee. (Assurance number A3158-01). Every effort to eliminate animal pain and distress through the use of anesthesia, analgesics or tranquilizers was made.

### Mice

C57Bl/6 mice and B6-Ly5.2 congenic mice were purchased from The Jackson Laboratory (Bar Harbor, ME). Rag-2-deficient (Rag2^−/−^) mice were purchased from Taconic Farms, Inc. TLR2-deficient (TLR2KO) mice were developed by S. Akira and colleagues [66]. TLR2KO mice were bred and maintained under pathogen-free conditions at the transgenic animal facility at the UMDNJ-NJMS. *M. tuberculosis*-infected mice were housed in the BSL3 facility at the Public Health Research Institute at UMDNJ-NJMS. Animal protocols used in this study were approved by the UMDNJ Institutional Animal Care and Use Committee.

### Mouse aerosol infections

The virulent Erdman strain (Trudeau Institute, Saranac Lake, NY) of *M. tuberculosis* was used for all infections. Bacterial stocks were generated by initial passage in C57Bl/6 mice. Bacterial colonies obtained from lung homogenates were grown in 7H9 media until mid-log phase, and the culture was stored in aliquots at −80°C. The stock titer was determined by plating 10-fold serial dilutions on Middlebrook 7H11 selective medium (Difco). Female mice (age 6–8 weeks) were infected via the respiratory route using a closed-air aerosolization system from In-TOX Products or the Inhalation exposure System from Glas-Col. Mice were exposed for 20 minutes to nebulized bacteria at a density optimized to deliver a standard low dose of around 100 CFU (unless otherwise indicated). For all infections, the actual infection dose was determined by plating total lung homogenates from a minimum of 2 mice on Middlebrook 7H11 plates at 24 hours after aerosol exposure.

### Analysis of tissues

Lungs and spleens were harvested at indicated time points post infection. The right superior lobe of the lung was used for determining bacterial burden. The right lower lobe was reserved for histological studies. The right middle lobe was reserved for protein determination. The postcaval lobe was reserved for tissue gene expression. The remaining lung tissue was cut into small pieces and digested with 2 mg/ml collagenase D (Roche) for 30 minutes at 37°C. The digestion was stopped by adding 10 mM EDTA. The digested tissue was forced through a 40 µm cell strainer (BD Falcon) to obtain single cell suspensions. Spleen tissues were processed similarly, but without collagenase digestion. Red blood cells were lysed using ACK lysing buffer (Quality Biological, Inc). The number of viable cells obtained per tissue was determined by trypan blue dye exclusion.

### Determination of bacterial burden

Lung tissue was homogenized in PBS containing 0.05% Tween-80. Total CFU per lung was determined by plating 10-fold serial dilutions on Middlebrook 7H11 plates. CFU were counted after 21 days of incubation at 37°C.

### Histopathology and immunohistochemistry

Lung tissue was fixed in 4% paraformaldehyde in PBS for four days, followed by paraffin embedding. For histopathological analysis, five to seven micrometer sections were cut and stained using a standard H&E protocol. For visualization of acid-fast bacilli, tissue sections were stained using the Ziehl-Neelsen method.

For immunohistochemical detection of Foxp3^+^ cells, tissue samples were de-paraffinized with xylene and rehydrated with ethanol gradations and water. The samples were subjected to heat-induced antigen retrieval by microwave warming using 10 mM citrate buffer (pH 6.0). Endogenous peroxidase activity was blocked using 0.3% hydrogen peroxide and then subsequently blocked with 1× PowerBlock (BioGenex). PBS containing 0.05% Tween-20 was used to wash tissues in between steps. For each sample, serial sections were incubated with the primary anti-mouse/rat Foxp3 antibody (clone FJK-16s; eBioscience) at a 1∶250 dilution or with isotype control (BioLegend) at the same concentration. Sections were subsequently incubated with biotinylated secondary antibody (1∶100 Vector Laboratories). Streptavidin horseradish peroxidase (BioGenex) was used to label the secondary antibody for immunodetection by DAB chromogen (BioGenex). After counterstaining with Mayer's hematoxylin (BioGenex), the samples were dehydrated with ethanol gradations, dipped in xylene, and mounted using Permount (Fisher Scientific).

For quantitation of involved lung area, photomicrographs of H&E stained lung sections were captured using a 5× objective lens. A 546 point grid overlay was superimposed onto each image using Image-Pro Discovery Software, and the numbers of points hitting areas of granulomatous infiltration were counted. The percentage of affected lung tissue was calculated as number of involved points/total points per section ×100.

### Flow cytometry

Single cell suspensions were stained at saturating conditions using specific monoclonal antibodies. All mAbs were directly conjugated to one of the following fluorochromes: Alexa Fluor 488, FITC, PE, PerCpCy5.5, PE-Cy7, APC, or Alexa Fluor700. Isotype controls were included for each. The following mAbs were used in the studies: CD4 (clone RM4–5), CD8 (clone 53-6.7), Foxp3 (clone FJK-16s), CD11c (clone HL3), CD11b (clone M1/70), Gr-1 (Ly-6G and Ly-6C; clone RB6-8C5), CD45.1 (clone A20), CD45.2 (clone 104). Abs to Foxp3, CD45.1, and CD45.2 was purchased from eBioscience. The remaining Abs were purchased from BD Biosciences. For surface staining, cells were re-suspended in FACS buffer (1× PBS +2% fetal calf serum and 0.09% sodium azide) containing a cocktail of mAbs against proteins of interest. For Foxp3 staining, surface staining was performed, followed by fixation, permeabilization, and intracellular staining of Foxp3 according to the manufacturer's protocol (Ebioscience). Following surface or intracytoplasmic staining, samples were fixed in 4% paraformaldehyde for 30 minutes and then acquired on a FACSCalibur or LSRII (BD Biosciences). Analysis was performed using FlowJo software (Tree Star, Inc.). Gating was based on isotype controls.

### ELISPOT assay

ELISPOT assay to detect the frequency of Mtb-specific IFNγ producing cells was performed as described previously [67]. 96-well MultiScreen HTS filter plates (Millipore) were coated with 8 µg/ml anti-IFNγ antibody (clone R4-6A2, BD Biosciences). Single cell suspensions from lungs were seeded at 0.25×10^5^, 0.5×10^5^, and 1×10^5^ cells per well. Cells were restimulated with Mtb-infected bone marrow-derived dendritic cells (3 MOI, overnight) at a ratio of 1∶2, or uninfected BMDCs as a control. The cultures were supplemented with IL-2 at 20 U/ml. The cells were co-cultured for 40 hr at 37°C. The plates were subsequently washed with PBS containing 0.05% Tween-20 and treated sequentially with biotinylated secondary antibody (Clone XMG1.2, BD Biosciences), ELISPOT streptavidin-HRP (BD Biosciences), and the HRP substrate 3-amino-9-ethyl-carbazole (Sigma). Spot-forming units were enumerated using an ELISPOT plate reader (Cellular Technology).

### Preparation of bone marrow-derived dendritic cells

Bone marrow-derived dendritic cells (BMDCs) were prepared as described previously [68]. Briefly, bone marrow cells were flushed from the femurs and tibiae of mice with PBS containing penicillin and streptomycin (100 U/ml each). Red blood cell lysis was performed using ACK lysing buffer. 2×10^6^ bone marrow cells were seeded into 10 cm Petri dishes in 10 ml RPMI-1640 media (Mediatech, Inc.) containing 10% defined FBS (HyClone Laboratories, Logan, UT) and supplemented with penicillin (100 U/ml), streptomycin (100 µg/ml), glutamine (2 mM), β-ME (50 µM), and 10% conditioned medium from murine GM-CSF-secreting X63 cells. On Day 3, an additional 10-ml complete medium containing GM-CSF was added to the cultures. On Day 6, the cultures were fed by changing fifty percent of the culture medium. Non-adherent cells were harvested on day 8.

### Adoptive transfer into Rag2^−/−^ mice

The MACS Regulatory T cell isolation kit (Miltenyi Biotec) was used to separate CD4^+^CD25^−^ and CD4^+^CD25^+^ lymphocytes from spleens and peripheral lymph nodes (axillary and inguinal) of naïve WT congenic B6-Ly5.2 mice (CD45.1^+^) and TLR2KO mice (CD45.2^+^). For adoptive transfer, a mixture of 2×10^6^ CD4^+^CD25^−^ (naïve conventional T cells) and 2×10^5^ CD4^+^CD25^+^ (natural regulatory T cells) in PBS were transferred into Rag2^−/−^ mice via retro-orbital injection. Group I received WT CD4^+^CD25^−^ and TLR2KO CD4^+^CD25^+^ cells, and group II received TLR2KO CD4^+^CD25^−^ and WT CD4^+^CD25^+^ cells. One day after transfer, recipient mice were infected with a low dose aerosol of Mtb. At 4 and 9 weeks post-infection, recipient mice were euthanized and lungs and spleens were used for analysis.

### RNA isolation and real-time PCR

Lung lobes were homogenized in 1 ml of TRIzol reagent in lysing matrix D tubes (MP Biomedicals) using a FastPrep homogenizer (MP Biomedicals). Samples were immediately stored at −80°C following lysis in TRIzol. Total RNA was extracted via the manufacturer's TRIzol/chloroform method and purified using RNeasy columns (Qiagen). Total RNA was reverse transcribed using Superscript II RT (Invitrogen). Real-time PCR was performed using the Mx3000P system (Stratagene). TaqMan gene expression assay (Applied Biosystems) for IL-17A and β-actin were used to determine relative IL-17 expression. Relative gene expression was determined by the ΔΔCt calculation^t^, where ΔCt = Ct (gene of interest) – Ct (normalizer = β-actin) and the ΔΔCt = ΔCt (sample) – ΔCt (calibrator). Total RNA from uninfected lungs was used as calibrator. Baseline gene expression from uninfected WT and TLR2KO was equivalent.

### Adoptive transfer of macrophages

Peritoneal exudate macrophages (PEM) were prepared and adoptively transferred as described previously [69]. Briefly, PEM from WT and TLR2KO mice (5 mice/group) were elicited by intra-peritoneal injection of 2 mls of sterile thioglycollate broth 5 days before peritoneal lavage. PEM from each group of mice were pooled and TLR2KO mice received 2.5×10^6^ WT or TLR2KO macrophages via the intra-tracheal route. One day after transfer, recipient mice were infected with a low dose aerosol of Mtb.

## Supporting Information

Figure S1 **Absence of TLR2 results in increased lung bacterial burden without affecting dissemination.** Acid-fast staining to visualize bacilli in lung tissue sections was performed using the Ziehl-Neelsen method (A). Formalin-fixed, paraffin-embedded lung tissue was obtained at 18 weeks following infection. Photomicrographs were taken at 40× magnification. Bacterial burden in spleens of WT and TLR2KO mice (B) following aerosol infection with approximately 150 CFU of Mtb was determined by plating serial dilutions of spleen homogenates onto 7H11 agar plates. Each time point includes 4–5 mice per group. Data are presented as mean CFU counts ± SEM. No significant differences in CFU were observed between WT and TLR2KO mice.
(TIF)

Figure S2 **Absence of TLR2 results in increased cellular recruitment to the lungs during chronic stages of infection.** Lungs were harvested from WT and TLR2KO mice, and single cell suspensions were prepared at the indicated time points after Mtb challenge. The total number of viable cells in the lungs was determined by trypan blue exclusion method (A). Lung cells (1×10^6^) were stained with antibodies against CD4, CD8, CD11c, CD11b, and Gr-1, and then analyzed by flow cytometry. The indicated populations were gated out of total live cells in the lungs. The absolute numbers of cells in each population in the lungs was determined by calculating the percentage of gated cells multiplied by total lung cell number. Recruitment of CD4 and CD8 T cells (A), and CD11c, CD11b, and Gr-1 cells (B) was determined in two different experiments. * = p<0.05, ** = p<0.01, *** = p<0.001.
(TIF)

Figure S3 **Gating strategy for quantitating Foxp3^+^ cells.** Lungs and spleens were harvested from WT and TLR2KO mice, and single cell suspensions were prepared at the indicated time points after Mtb challenge. Cells were stained with antibodies against CD4, followed by intracellular staining for Foxp3. Representative gating of Foxp3 out of CD4 in the lungs of WT and TLR2KO mice is shown.
(TIF)

Figure S4 **Foxp3-expressing cells in the lungs at 4 weeks post-Mtb infection.** Formalin-fixed, paraffin-embedded lung tissue was obtained at 4 weeks following infection. Serial sections showing areas of granulomatous inflammation in WT (A–D) and TLR2KO (E–H) mice are shown. Sections were stained with H&E (A and E) or with anti-Foxp3 (B and F). In sections stained with anti-Foxp3, areas within the lung parenchyma (C and G) and perivascular/peribronchiolar areas (D and H) are shown at higher magnification. Brown pinpoint staining indicative of Foxp3^+^ staining was not observed in serial sections stained with isotype control (not shown). Photomicrographs were taken at 10× (A, B, E, and F) and at 40× (C, D, G, and H) original magnification. Sections are representative of 5 mice per group.
(TIF)

Figure S5 **Adoptive transfer of congenic T cell populations into Rag2^−/−^ mice.** Rag2^−/−^ mice were reconstituted with combinations of WT and TLR2KO CD4^+^CD25^−^ and CD4^+^CD25^+^ T cells one day prior to aerosol Mtb infection. Schematic of the adoptive transfer is shown (A). Flow cytometric analysis of the injected populations in the lungs (B, D, and F) and spleens (C, E, and G) was performed to evaluate Foxp3 expression during Mtb infection. Single cell suspensions were stained with antibodies against CD4, CD45.1, and CD45.2, followed by intracellular staining for Foxp3. Lymphocytes were gated on, followed by gating on the CD4^+^ population. The top panels (B and C) show CD45.1 and CD45.2 populations out of the gated CD4^+^ cells in lungs and spleens, respectively. Lower histograms (D and E) show Foxp3 expression out of the gated CD45.1^+^CD4^+^ and CD45.2^+^CD4^+^ cells. Foxp3 expression in the gated populations, as shown in histograms, is represented quantitatively (data from 6 mice) in panels F and G. The injected CD4^+^CD25^+^ population is blue for group I and red for group II. The injected CD4^+^CD25^−^ population is black for both groups. Representative plots at 4 weeks post-infection are shown.
(TIF)

## References

[1] Sutcliffe IC, Harrington DJ (2004) Lipoproteins of Mycobacterium tuberculosis: an abundant and functionally diverse class of cell envelope components. FEMS Microbiol Rev 28: 645–659. 15539077 10.1016/j.femsre.2004.06.002

[2] Drage MG, Tsai HC, Pecora ND, Cheng TY, Arida AR, et al (2010) Mycobacterium tuberculosis lipoprotein LprG (Rv1411c) binds triacylated glycolipid agonists of Toll-like receptor 2. Nat Struct Mol Biol 17: 1088–1095. 20694006 10.1038/nsmb.1869 PMC2933325

[3] Pecora ND, Gehring AJ, Canaday DH, Boom WH, Harding CV (2006) Mycobacterium tuberculosis LprA is a lipoprotein agonist of TLR2 that regulates innate immunity and APC function. J Immunol 177: 422–429. 16785538 10.4049/jimmunol.177.1.422

[4] Underhill DM, Ozinsky A, Smith KD, Aderem A (1999) Toll-like receptor-2 mediates mycobacteria-induced proinflammatory signaling in macrophages. Proc Natl Acad Sci 96: 14459–14463. 10588727 10.1073/pnas.96.25.14459 PMC24458

[5] Means TK, Wang S, Lien E, Yoshimura A, Golenbock DT, et al (1999) Human toll-like receptors mediate cellular activation by Mycobacterium tuberculosis. J Immunol 163: 3920–3927. 10490993

[6] Hertz CJ, Kiertscher SM, Godowski PJ, Bouis DA, Norgard MV, et al (2001) Microbial lipopeptides stimulate dendritic cell maturation via Toll-like receptor 2. J Immunol 166: 2444–2450. 11160304 10.4049/jimmunol.166.4.2444

[7] Teixeira-Coelho M, Cruz A, Carmona J, Sousa C, Ramos-Pereira D, et al (2011) TLR2 deficiency by compromising p19 (IL-23) expression limits Th 17 cell responses to Mycobacterium tuberculosis. Int Immunol 23: 89–96. 21156751 10.1093/intimm/dxq459

[8] Thoma-Uszynski S, Stenger S, Takeuchi O, Ochoa MT, Engele M, et al (2001) Induction of Direct Antimicrobial Activity Through Mammalian Toll-Like Receptors. Science 291: 1544–1547. 11222859 10.1126/science.291.5508.1544

[9] Liu PT, Stenger S, Li H, Wenzel L, Tan BH, et al (2006) Toll-like receptor triggering of a vitamin D-mediated human antimicrobial response. Science 311: 1770–1773. 16497887 10.1126/science.1123933

[10] Jang S, Uematsu S, Akira S, Salgame P (2004) IL-6 and IL-10 induction from dendritic cells in response to Mycobacterium tuberculosis is predominantly dependent on TLR2-mediated recognition. J Immunol 173: 3392–3397. 15322203 10.4049/jimmunol.173.5.3392

[11] Noss EH, Pai RK, Sellati TJ, Radolf JD, Belisle J, et al (2001) Toll-like receptor 2-dependent inhibition of macrophage class II MHC expression and antigen processing by 19-kDa lipoprotein of Mycobacterium tuberculosis. J Immunol 167: 910–918. 11441098 10.4049/jimmunol.167.2.910

[12] Gehring AJ, Dobos KM, Belisle JT, Harding CV, Boom WH (2004) Mycobacterium tuberculosis LprG (Rv1411c): a novel TLR-2 ligand that inhibits human macrophage class II MHC antigen processing. J Immunol 173: 2660–2668. 15294983 10.4049/jimmunol.173.4.2660

[13] Harding CV, Boom WH (2010) Regulation of antigen presentation by Mycobacterium tuberculosis: a role for Toll-like receptors. Nat Rev Microbiol 8: 296–307. 20234378 10.1038/nrmicro2321 PMC3037727

[14] Fortune SM, Solache A, Jaeger A, Hill PJ, Belisle JT, et al (2004) Mycobacterium tuberculosis inhibits macrophage responses to IFN-gamma through myeloid differentiation factor 88-dependent and -independent mechanisms. J Immunol 172: 6272–6280. 15128816 10.4049/jimmunol.172.10.6272

[15] Reiling N, Holscher C, Fehrenbach A, Kroger S, Kirschning CJ, et al (2002) Cutting edge: Toll-like receptor (TLR)2- and TLR4-mediated pathogen recognition in resistance to airborne infection with Mycobacterium tuberculosis. J Immunol 169: 3480–3484. 12244136 10.4049/jimmunol.169.7.3480

[16] Sugawara I, Yamada H, Li C, Mizuno S, Takeuchi O, et al (2003) Mycobacterial infection in TLR2 and TLR6 knockout mice. Microbiol Immuno 47: 327–336. 10.1111/j.1348-0421.2003.tb03404.x 12825894

[17] Drennan MB, Nicolle D, Quesniaux VJ, jacobs M, Allie N, et al (2004) Toll-Like receptor 2-deficient mice succumb to Mycobacterium tuberculosis infection. Am J pathol 164: 49–57. 14695318 10.1016/S0002-9440(10)63095-7 PMC1602241

[18] McBride A, Bhatt K, Salgame P (2011) Development of a Secondary Immune Response to Mycobacterium tuberculosis Is Independent of Toll-Like Receptor 2. Infect Immun 79: 1118–1123. 21173309 10.1128/IAI.01076-10 PMC3067489

[19] Ogus AC, Yoldas B, Ozdemir T, Uguz A, Olcen S, et al (2004) The Arg753GLn polymorphism of the human toll-like receptor 2 gene in tuberculosis disease. Eur Respir J 23: 219–223. 14979495 10.1183/09031936.03.00061703

[20] Ben-Ali M, Barbouche MR, Bousnina S, Chabbou A, Dellagi K (2004) Toll-like receptor 2 Arg677Trp polymorphism is associated with susceptibility to tuberculosis in Tunisian patients. Clin Diagn Lab Immunol 11: 625–626. 15138193 10.1128/CDLI.11.3.625-626.2004 PMC404573

[21] Velez DR, Wejse C, Stryjewski ME, Abbate E, Hulme WF, et al (2010) Variants in toll-like receptors 2 and 9 influence susceptibility to pulmonary tuberculosis in Caucasians, African-Americans, and West Africans. Hum Genet 127: 65–73. 19771452 10.1007/s00439-009-0741-7 PMC2902366

[22] Yim JJ, Lee HW, Lee HS, Kim YW, Han SK, et al (2006) The association between microsatellite polymorphisms in intron II of the human Toll-like receptor 2 gene and tuberculosis among Koreans. Genes Immun 7: 150–155. 16437124 10.1038/sj.gene.6364274

[23] Hawn TR, Misch EA, Dunstan SJ, Thwaites GE, Lan NT, et al (2007) A common human TLR1 polymorphism regulates the innate immune response to lipopeptides. Eur J Immunol 37: 2280–2289. 17595679 10.1002/eji.200737034

[24] Lazarevic V, Myers AJ, Scanga CA, Flynn JL (2003) CD40, but not CD40L, is required for the optimal priming of T cells and control of aerosol M. tuberculosis infection. Immunity 19: 823–835. 14670300 10.1016/s1074-7613(03)00324-8

[25] Flynn JL (2006) Lessons from experimental Mycobacterium tuberculosis infections. Microbes Infect 8: 1179–1188. 16513383 10.1016/j.micinf.2005.10.033

[26] Scott-Browne JP, Shafiani S, Tucker-Heard G, Ishida-Tsubota K, Fontenot JD, et al (2007) Expansion and function of Foxp3-expressing T regulatory cells during tuberculosis. J Exp Med 204: 2159–2169. 17709423 10.1084/jem.20062105 PMC2118702

[27] Quinn KM, McHugh RS, Rich FJ, Goldsack LM, de Lisle GW, et al (2006) Inactivation of CD4+ CD25+ regulatory T cells during early mycobacterial infection increases cytokine production but does not affect pathogen load. Immunol Cell Biol 84: 467–474. 16869940 10.1111/j.1440-1711.2006.01460.x

[28] Kursar M, Koch M, Mittrucker HW, Nouailles G, Bonhagen K, et al (2007) Cutting Edge: Regulatory T cells prevent efficient clearance of Mycobacterium tuberculosis. J Immunol 178: 2661–2665. 17312107 10.4049/jimmunol.178.5.2661

[29] Liu H, Komai-Koma M, Xu D, Liew FY (2006) Toll-like receptor 2 signaling modulates the functions of CD4+ CD25+ regulatory T cells. Proc Natl Acad Sci U S A 103: 7048–7053. 16632602 10.1073/pnas.0601554103 PMC1444884

[30] Sutmuller RP, den Brok MH, Kramer M, Bennink EJ, Toonen LW, et al (2006) Toll-like receptor 2 controls expansion and function of regulatory T cells. J Clin Invest 116: 485–494. 16424940 10.1172/JCI25439 PMC1332026

[31] Sutmuller RP, Morgan ME, Netea MG, Grauer O, Adema GJ (2006) Toll-like receptors on regulatory T cells: expanding immune regulation. Trends Immunol 27: 387–393. 16814607 10.1016/j.it.2006.06.005

[32] Netea MG, Sutmuller R, Hermann C, Van der Graaf CA, Van der Meer JW, et al (2004) Toll-like receptor 2 suppresses immunity against Candida albicans through induction of IL-10 and regulatory T cells. J Immunol 172: 3712–3718. 15004175 10.4049/jimmunol.172.6.3712

[33] Maglione PJ, Xu J, Chan J (2007) B cells moderate inflammatory progression and enhance bacterial containment upon pulmonary challenge with Mycobacterium tuberculosis. J Immunol 178: 7222–7234. 17513771 10.4049/jimmunol.178.11.7222

[34] Brown J, Wang H, Hajishengallis GN, Martin M (2011) TLR-signaling networks: an integration of adaptor molecules, kinases, and cross-talk. J Dent Res 90: 417–427. 20940366 10.1177/0022034510381264 PMC3075579

[35] Iellem A, Mariani M, Lang R, Recalde H, Panina-Bordignon P, et al (2001) Unique chemotactic response profile and specific expression of chemokine receptors CCR4 and CCR8 by CD4(+)CD25(+) regulatory T cells. J Exp Med 194: 847–853. 11560999 10.1084/jem.194.6.847 PMC2195967

[36] Hirahara K, Liu L, Clark RA, Yamanaka K, Fuhlbrigge RC, et al (2006) The majority of human peripheral blood CD4+CD25highFoxp3+ regulatory T cells bear functional skin-homing receptors. J Immunol 177: 4488–4494. 16982885 10.4049/jimmunol.177.7.4488

[37] Mizukami Y, Kono K, Kawaguchi Y, Akaike H, Kamimura K, et al (2008) CCL17 and CCL22 chemokines within tumor microenvironment are related to accumulation of Foxp3+ regulatory T cells in gastric cancer. Int J Cancer 122: 2286–2293. 18224687 10.1002/ijc.23392

[38] Curiel TJ, Coukos G, Zou L, Alvarez X, Cheng P, et al (2004) Specific recruitment of regulatory T cells in ovarian carcinoma fosters immune privilege and predicts reduced survival. Nat Med 10: 942–949. 15322536 10.1038/nm1093

[39] Haas J, Schopp L, Storch-Hagenlocher B, Fritzsching B, Jacobi C, et al (2008) Specific recruitment of regulatory T cells into the CSF in lymphomatous and carcinomatous meningitis. Blood 111: 761–766. 17967942 10.1182/blood-2007-08-104877

[40] Yang ZZ, Novak AJ, Stenson MJ, Witzig TE, Ansell SM (2006) Intratumoral CD4+CD25+ regulatory T-cell-mediated suppression of infiltrating CD4+ T cells in B-cell non-Hodgkin lymphoma. Blood 107: 3639–3646. 16403912 10.1182/blood-2005-08-3376 PMC1895773

[41] Mailloux AW, Young MR (2009) NK-dependent increases in CCL22 secretion selectively recruits regulatory T cells to the tumor microenvironment. J Immunol 182: 2753–2765. 19234170 10.4049/jimmunol.0801124 PMC3337694

[42] Koch MA, Tucker-Heard G, Perdue NR, Killebrew JR, Urdahl KB, et al (2009) The transcription factor T-bet controls regulatory T cell homeostasis and function during type 1 inflammation. Nat Immunol 10: 595–602. 19412181 10.1038/ni.1731 PMC2712126

[43] Beatty WL, Russell DG (2000) Identification of mycobacterial surface proteins released into subcellular compartments of infected macrophages. Infect Immun 68: 6997–7002. 11083824 10.1128/iai.68.12.6997-7002.2000 PMC97809

[44] Bhatnagar S, Schorey JS (2007) Exosomes released from infected macrophages contain Mycobacterium avium glycopeptidolipids and are proinflammatory. J Biol Chem 282: 25779–25789. 17591775 10.1074/jbc.M702277200 PMC3636815

[45] Prados-Rosales R, Baena A, Martinez LR, Luque-Garcia J, Kalscheuer R, et al (2011) Mycobacteria release active membrane vesicles that modulate immune responses in a TLR2-dependent manner in mice. J Clin Invest 121: 1471–1483. 21364279 10.1172/JCI44261 PMC3069770

[46] Ribeiro-Rodrigues R, Resende Co T, Rojas R, Toossi Z, Dietze R, et al (2006) A role for CD4+CD25+ T cells in regulation of the immune response during human tuberculosis. Clin Exp Immunol 144: 25–34. 16542361 10.1111/j.1365-2249.2006.03027.x PMC1809641

[47] Guyot-Revol V, Innes JA, Hackforth S, Hinks T, Lalvani A (2006) Regulatory T cells are expanded in blood and disease sites in patients with tuberculosis. Am J Respir Crit Care Med 173: 803–810. 16339919 10.1164/rccm.200508-1294OC

[48] Hougardy JM, Place S, Hildebrand M, Drowart A, Debrie AS, et al (2007) Regulatory T cells depress immune responses to protective antigens in active tuberculosis. Am J Respir Crit Care Med 176: 409–416. 17541018 10.1164/rccm.200701-084OC

[49] Shafiani S, Tucker-Heard G, Kariyone A, Takatsu K, Urdahl KB (2010) Pathogen-specific regulatory T cells delay the arrival of effector T cells in the lung during early tuberculosis. J Exp Med 207: 1409–1420. 20547826 10.1084/jem.20091885 PMC2901066

[50] Green AM, Mattila JT, Bigbee CL, Bongers KS, Lin PL, et al (2010) CD4(+) Regulatory T Cells in a Cynomolgus Macaque Model of Mycobacterium tuberculosis Infection. J Infect Dis 202: 533–541. 20617900 10.1086/654896 PMC3683560

[51] Chen CY, Huang D, Yao S, Halliday L, Zeng G, et al (2012) IL-2 simultaneously expands Foxp3+ T regulatory and T effector cells and confers resistance to severe tuberculosis (TB): implicative Treg-T effector cooperation in immunity to TB. J Immunol 188: 4278–4288. 22474020 10.4049/jimmunol.1101291 PMC3412415

[52] Holscher C, Reiling N, Schaible UE, Holscher A, Bathmann C, et al (2008) Containment of aerogenic Mycobacterium tuberculosis infection in mice does not require MyD88 adaptor function for TLR2, −4 and −9. Eur J Immunol 38: 680–694. 18266299 10.1002/eji.200736458

[53] Reiling N, Ehlers S, Holscher C (2008) MyDths and un-TOLLed truths: sensor, instructive and effector immunity to tuberculosis. Immunol Lett 116: 15–23. 18191460 10.1016/j.imlet.2007.11.015

[54] Ichinohe T, Pang IK, Kumamoto Y, Peaper DR, Ho JH, et al (2011) Microbiota regulates immune defense against respiratory tract influenza A virus infection. Proc Natl Acad Sci U S A 108: 5354–5359. 21402903 10.1073/pnas.1019378108 PMC3069176

[55] Powrie F (2004) Immune regulation in the intestine: a balancing act between effector and regulatory T cell responses. Ann N Y Acad Sci 1029: 132–141. 15681752 10.1196/annals.1309.030

[56] Coombes JL, Maloy KJ (2007) Control of intestinal homeostasis by regulatory T cells and dendritic cells. Semin Immunol 19: 116–126. 17320411 10.1016/j.smim.2007.01.001

[57] Nandi B, Behar SM (2011) Regulation of neutrophils by interferon-gamma limits lung inflammation during tuberculosis infection. J Exp Med 208: 2251–2262. 21967766 10.1084/jem.20110919 PMC3201199

[58] Mayer-Barber KD, Andrade BB, Barber DL, Hieny S, Feng CG, et al (2011) Innate and adaptive interferons suppress IL-1alpha and IL-1beta production by distinct pulmonary myeloid subsets during Mycobacterium tuberculosis infection. Immunity 35: 1023–1034. 22195750 10.1016/j.immuni.2011.12.002 PMC3246221

[59] Mishra BB, Rathinam VA, Martens GW, Martinot AJ, Kornfeld H, et al (2013) Nitric oxide controls the immunopathology of tuberculosis by inhibiting NLRP3 inflammasome-dependent processing of IL-1beta. Nat Immunol 14: 52–60. 23160153 10.1038/ni.2474 PMC3721324

[60] Kozakiewicz L, Phuah J, Flynn J, Chan J (2013) The Role of B Cells and Humoral Immunity in Mycobacterium tuberculosis Infection. Adv Exp Med Biol 783: 225–250. 23468112 10.1007/978-1-4614-6111-1_12 PMC4184189

[61] Yang CT, Cambier CJ, Davis JM, Hall CJ, Crosier PS, et al (2012) Neutrophils exert protection in the early tuberculous granuloma by oxidative killing of mycobacteria phagocytosed from infected macrophages. Cell Host Microbe 12: 301–312. 22980327 10.1016/j.chom.2012.07.009 PMC3638950

[62] Lee WB, Kang JS, Yan JJ, Lee MS, Jeon BY, et al (2012) Neutrophils Promote Mycobacterial Trehalose Dimycolate-Induced Lung Inflammation via the Mincle Pathway. PLoS Pathog 8: e1002614. 22496642 10.1371/journal.ppat.1002614 PMC3320589

[63] Lowe DM, Redford PS, Wilkinson RJ, O'Garra A, Martineau AR (2012) Neutrophils in tuberculosis: friend or foe? Trends Immunol 33: 14–25. 22094048 10.1016/j.it.2011.10.003

[64] Volkman HE, Pozos TC, Zheng J, Davis JM, Rawls JF, et al (2010) Tuberculous granuloma induction via interaction of a bacterial secreted protein with host epithelium. Science 327: 466–469. 20007864 10.1126/science.1179663 PMC3125975

[65] Davis JM, Ramakrishnan L (2009) The role of the granuloma in expansion and dissemination of early tuberculous infection. Cell 136: 37–49. 19135887 10.1016/j.cell.2008.11.014 PMC3134310

[66] Takeuchi O, Hoshino K, Kawai T, Sanjo H, Takada H, et al (1999) Differential roles of TLR2 and TLR4 in recognition of gram-negative and gram-positive bacterial cell wall components. Immunity 11: 443–451. 10549626 10.1016/s1074-7613(00)80119-3

[67] Lazarevic V, Yankura DJ, DiVito SJ, Flynn JL (2005) Induction of Mycobacterium tuberculosis-specific primary and secondary T-cell responses in interleukin-15-deficient mice. Infect Immun 73: 2910–2922. 15845497 10.1128/IAI.73.5.2910-2922.2005 PMC1087383

[68] Hickman SP, Chan J, Salgame P (2002) Mycobacterium tuberculosis induces differential cytokine production from dendritic cells and macrophages with divergent effects on naive T cell polarization. J Immunol 168: 4636–4642. 11971012 10.4049/jimmunol.168.9.4636

[69] Potian JA, Rafi W, Bhatt K, McBride A, Gause WC, et al (2011) Preexisting helminth infection induces inhibition of innate pulmonary anti-tuberculosis defense by engaging the IL-4 receptor pathway. J Exp Med 208: 1863–1874. 21825018 10.1084/jem.20091473 PMC3171086

