# Supplementary figures and images for: Host Defense and Recruitment of Foxp3+ T Regulatory Cells to the Lungs in Chronic Mycobacterium tuberculosis Infection Requires Toll-like Receptor 2
Source: PLoS Pathog. 2013 Jun 13;9(6):e1003397. doi: 10.1371/journal.ppat.1003397 (PMC3681744; doi:10.1371/journal.ppat.1003397)

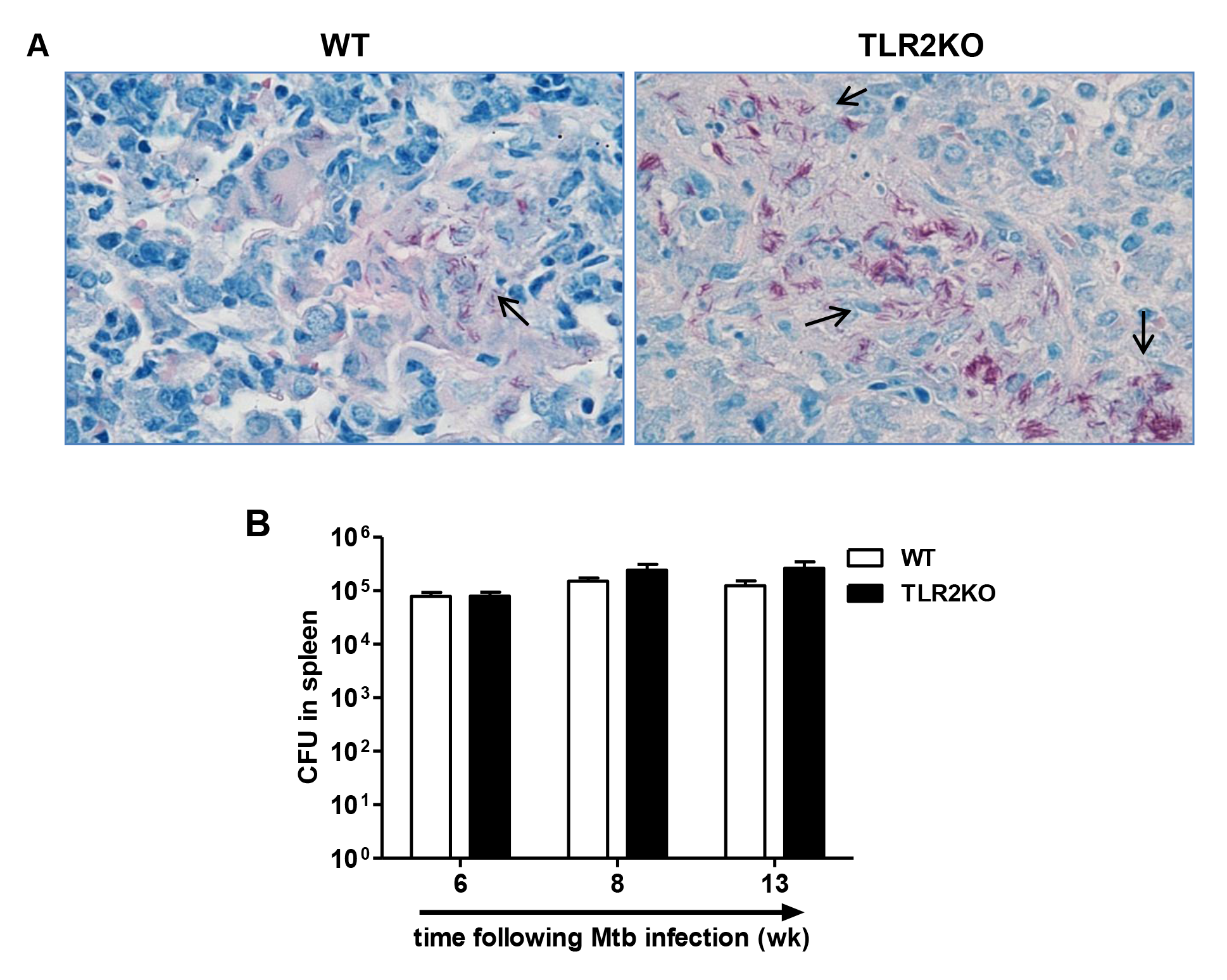

Supplement: Figure S1 — Absence of TLR2 results in increased lung bacterial burden without affecting dissemination. Acid-fast staining to visualize bacilli in lung tissue sections was performed using the Ziehl-Neelsen method (A). Formalin-fixed, paraffin-embedded lung tissue was obtained at 18 weeks following infection. Photomicrographs were taken at 40× magnification. Bacterial burden in spleens of WT and TLR2KO mice (B) following aerosol infection with approximately 150 CFU of Mtb was determined by plating serial dilutions of spleen homogenates onto 7H11 agar plates. Each time point includes 4–5 mice per group. Data are presented as mean CFU counts ± SEM. No significant differences in CFU were observed between WT and TLR2KO mice. (TIF) [file ppat.1003397.s001.tif]

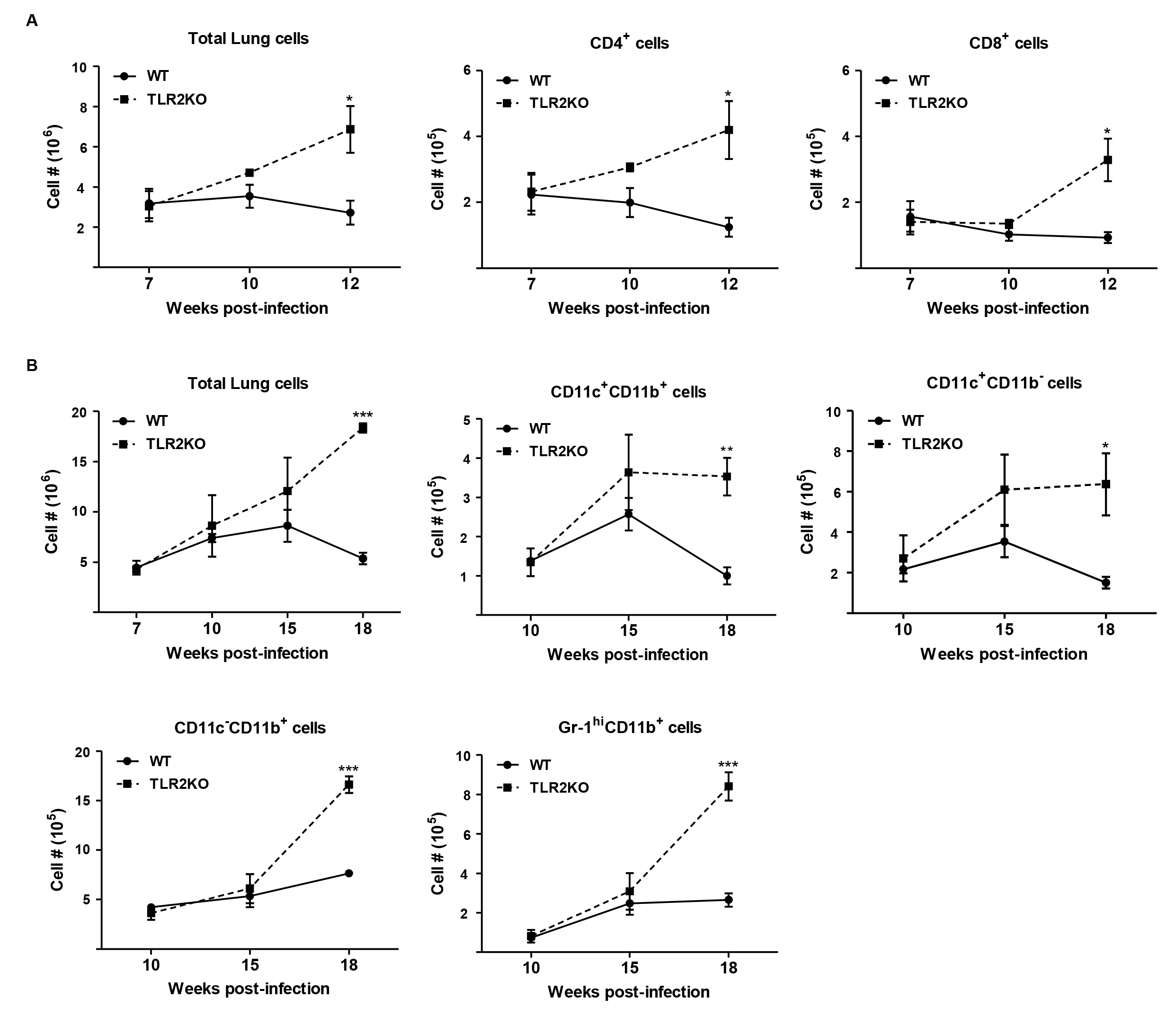

Supplement: Figure S2 — Absence of TLR2 results in increased cellular recruitment to the lungs during chronic stages of infection. Lungs were harvested from WT and TLR2KO mice, and single cell suspensions were prepared at the indicated time points after Mtb challenge. The total number of viable cells in the lungs was determined by trypan blue exclusion method (A). Lung cells (1×10^6^) were stained with antibodies against CD4, CD8, CD11c, CD11b, and Gr-1, and then analyzed by flow cytometry. The indicated populations were gated out of total live cells in the lungs. The absolute numbers of cells in each population in the lungs was determined by calculating the percentage of gated cells multiplied by total lung cell number. Recruitment of CD4 and CD8 T cells (A), and CD11c, CD11b, and Gr-1 cells (B) was determined in two different experiments. * = p<0.05, ** = p<0.01, *** = p<0.001. (TIF) [file ppat.1003397.s002.tif]

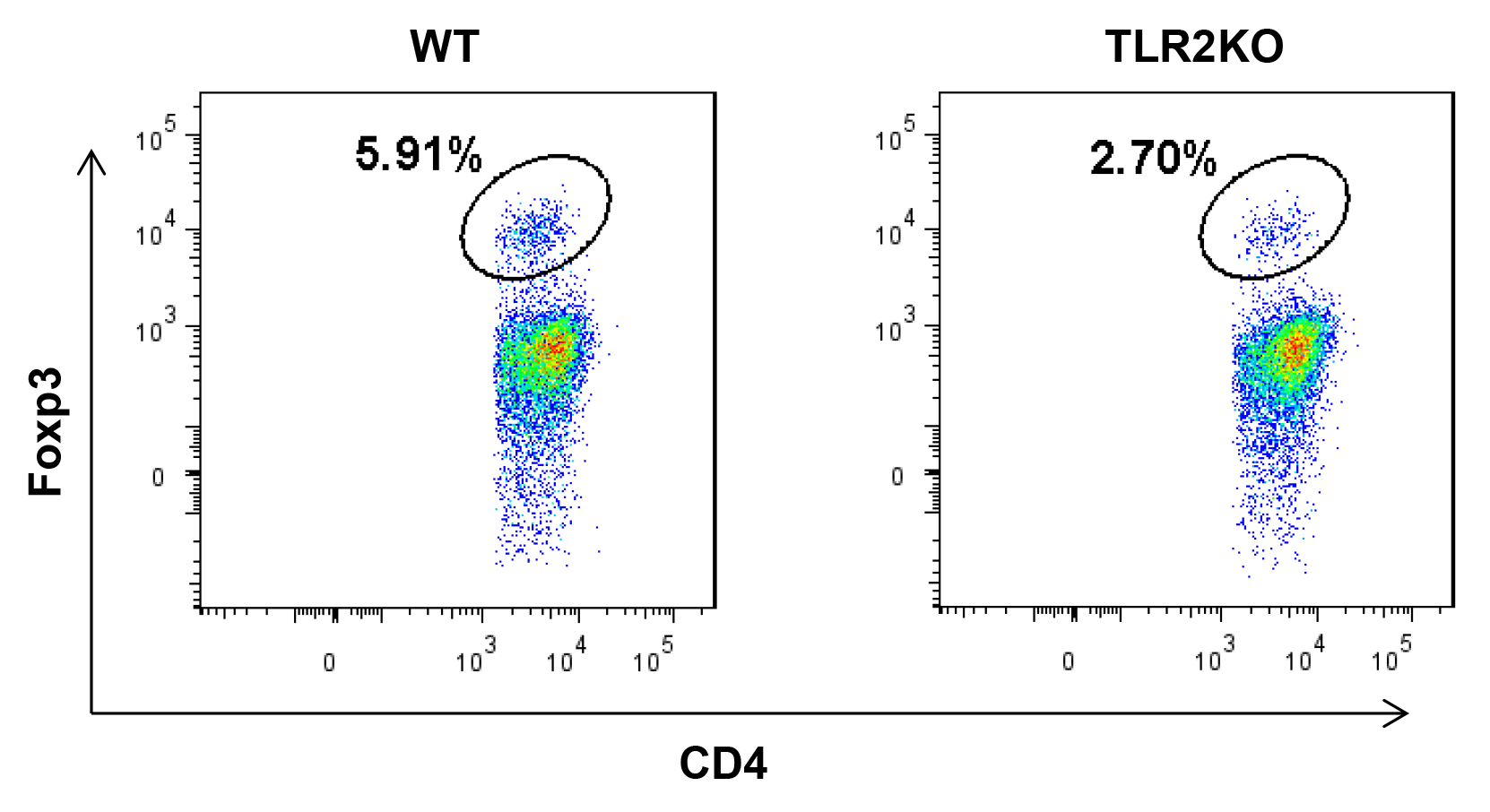

Supplement: Figure S3 — Gating strategy for quantitating Foxp3+ cells. Lungs and spleens were harvested from WT and TLR2KO mice, and single cell suspensions were prepared at the indicated time points after Mtb challenge. Cells were stained with antibodies against CD4, followed by intracellular staining for Foxp3. Representative gating of Foxp3 out of CD4 in the lungs of WT and TLR2KO mice is shown. (TIF) [file ppat.1003397.s003.tif]

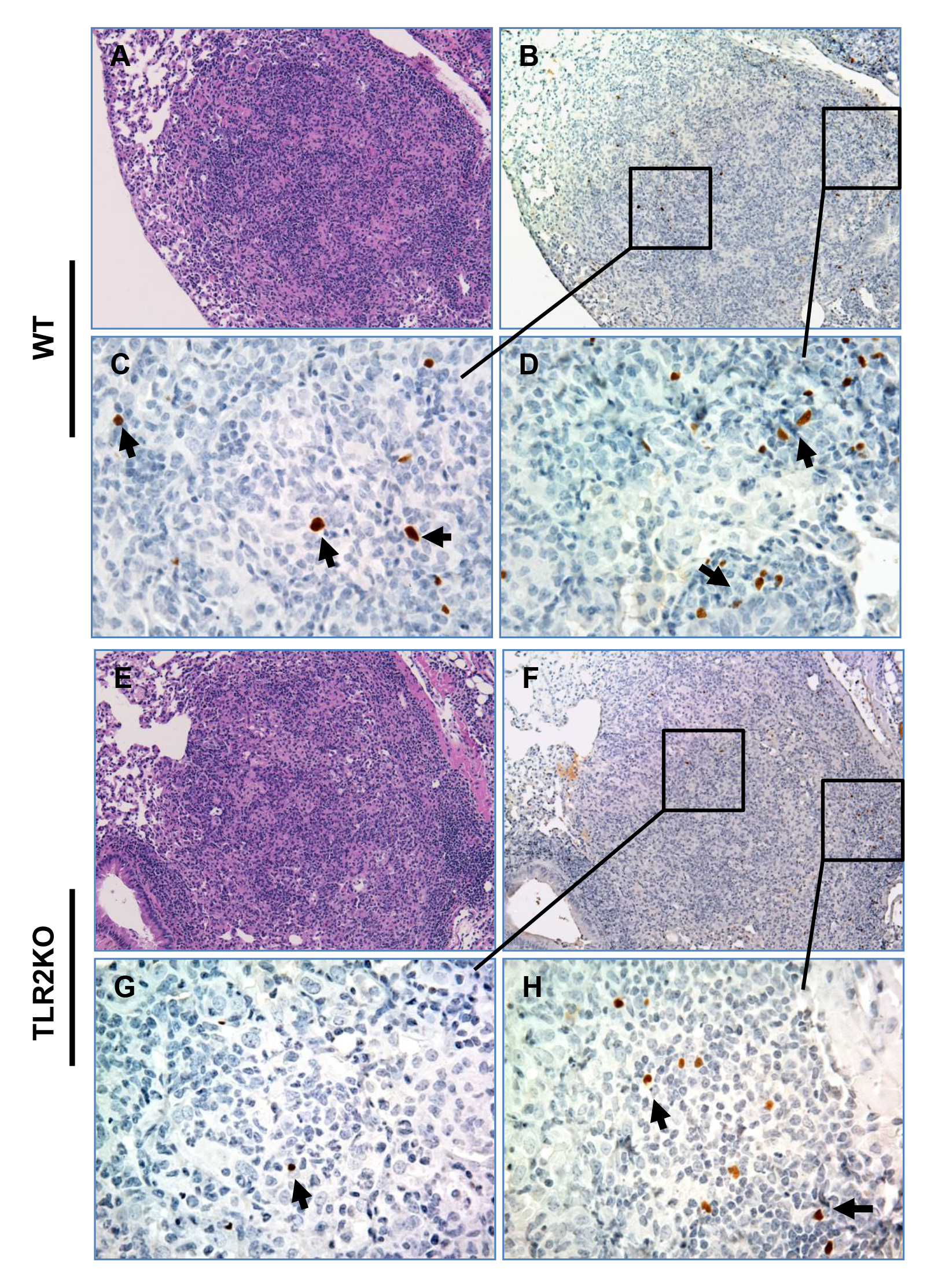

Supplement: Figure S4 — Foxp3-expressing cells in the lungs at 4 weeks post-Mtb infection. Formalin-fixed, paraffin-embedded lung tissue was obtained at 4 weeks following infection. Serial sections showing areas of granulomatous inflammation in WT (A–D) and TLR2KO (E–H) mice are shown. Sections were stained with H&E (A and E) or with anti-Foxp3 (B and F). In sections stained with anti-Foxp3, areas within the lung parenchyma (C and G) and perivascular/peribronchiolar areas (D and H) are shown at higher magnification. Brown pinpoint staining indicative of Foxp3+ staining was not observed in serial sections stained with isotype control (not shown). Photomicrographs were taken at 10× (A, B, E, and F) and at 40× (C, D, G, and H) original magnification. Sections are representative of 5 mice per group. (TIF) [file ppat.1003397.s004.tif]

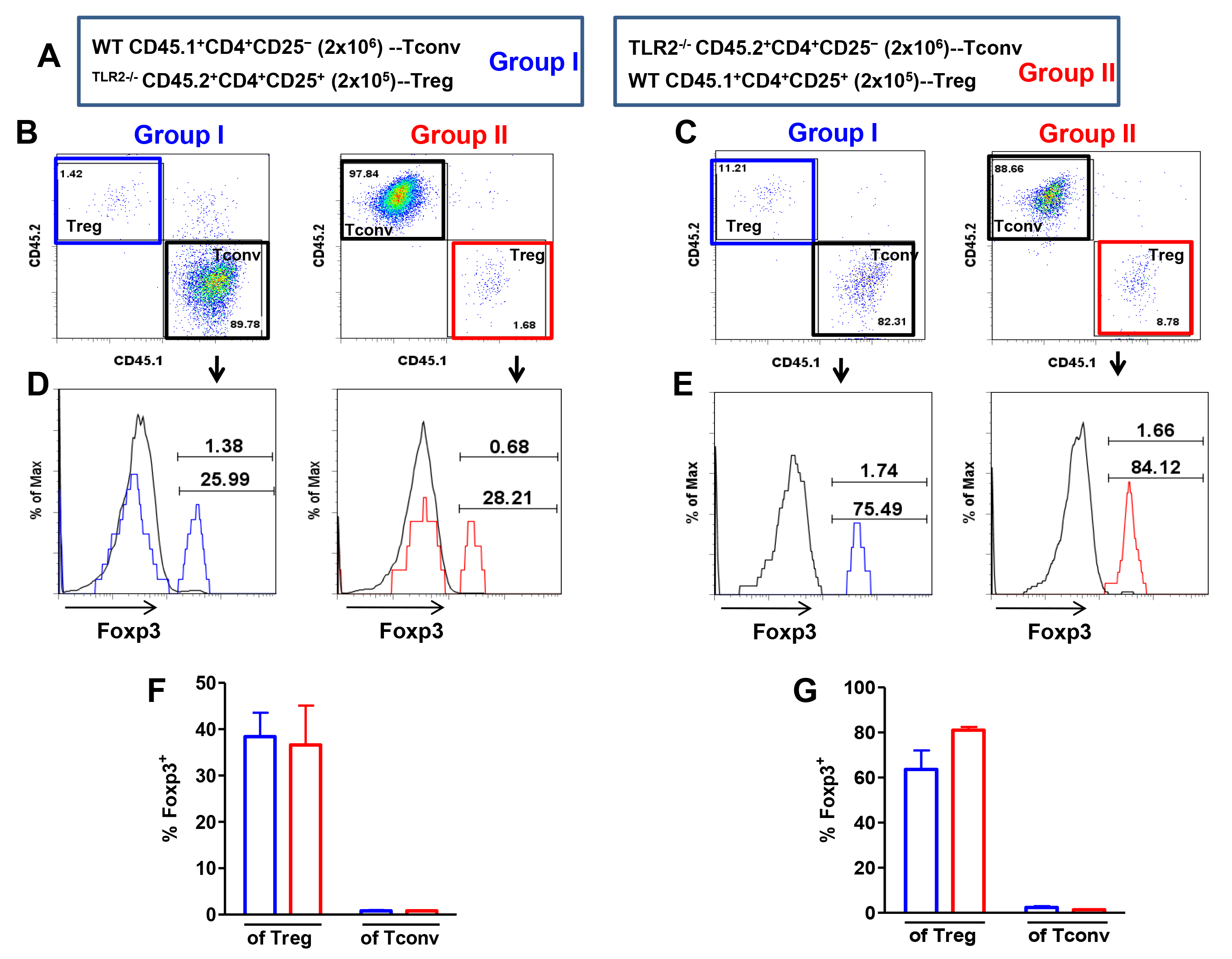

Supplement: Figure S5 — Adoptive transfer of congenic T cell populations into Rag2−/− mice. Rag2−/− mice were reconstituted with combinations of WT and TLR2KO CD4+CD25− and CD4+CD25+ T cells one day prior to aerosol Mtb infection. Schematic of the adoptive transfer is shown (A). Flow cytometric analysis of the injected populations in the lungs (B, D, and F) and spleens (C, E, and G) was performed to evaluate Foxp3 expression during Mtb infection. Single cell suspensions were stained with antibodies against CD4, CD45.1, and CD45.2, followed by intracellular staining for Foxp3. Lymphocytes were gated on, followed by gating on the CD4+ population. The top panels (B and C) show CD45.1 and CD45.2 populations out of the gated CD4+ cells in lungs and spleens, respectively. Lower histograms (D and E) show Foxp3 expression out of the gated CD45.1+CD4+ and CD45.2+CD4+ cells. Foxp3 expression in the gated populations, as shown in histograms, is represented quantitatively (data from 6 mice) in panels F and G. The injected CD4+CD25+ population is blue for group I and red for group II. The injected CD4+CD25− population is black for both groups. Representative plots at 4 weeks post-infection are shown. (TIF) [file ppat.1003397.s005.tif]
